# Metal Requirements for Building Electrical Grid Systems of Global Wind Power and Utility-Scale Solar Photovoltaic until 2050

**DOI:** 10.1021/acs.est.2c06496

**Published:** 2022-12-29

**Authors:** Zhenyang Chen, Rene Kleijn, Hai Xiang Lin

**Affiliations:** † Institute of Environmental Sciences (CML), Leiden University, Leiden2333 CC, The Netherlands; ‡ Delft Institute of Applied Mathematics, Delft University of Technology, Delft2628 CD, The Netherlands

**Keywords:** transmission infrastructure, power cable, renewable energy, mineral resources, material recycling

## Abstract

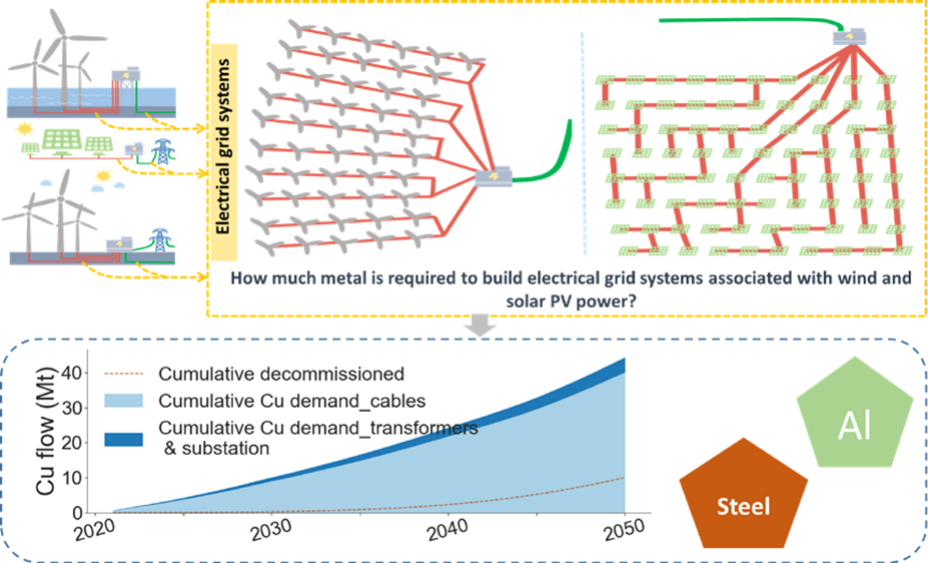

Wind and solar photovoltaic (PV) power form vital parts of the energy transition toward renewable energy systems. The rapid development of these two renewables represents an enormous infrastructure construction task including both power generation and its associated electrical grid systems, which will generate demand for metal resources. However, most research on material demands has focused on their power generation systems (wind turbines and PV panels), and few have studied the associated electrical grid systems. Here, we estimate the global metal demands for electrical grid systems associated with wind and utility-scale PV power by 2050, using dynamic material flow analysis based on International Energy Agency’s energy scenarios and the typical engineering parameters of transmission grids. Results show that the associated electrical grids require large quantities of metals: 27–81 Mt of copper cumulatively, followed by 20–67 Mt of steel and 11–31 Mt of aluminum. Electrical grids built for solar PV have the largest metal demand, followed by offshore and onshore wind. Power cables are the most metal-consuming electrical components compared to substations and transformers. We also discuss the decommissioning issue of electrical grids and their recovery potential. This study would deepen the understanding of the nexus between renewable energy, grid infrastructure, and metal resources.

## Abstract 2

Minimal research exists on estimating material requirements for grid infrastructure of wind and solar PV. This study quantifies metal requirements for materializing electrical grids related to wind and solar PV.

## 1 Introduction

The cumulative installed capacities of global wind and solar photovoltaic (PV) power have experienced rapid development, increasing by 12 times and 200 times, respectively, from 2003 to 2019.^1,2^ The enormous growth of these two renewables is primarily driven by the continuous cost reduction, as well as growing consensus on the energy transition for climate change mitigation.^3,4^ Wind and solar PV technologies are expected to continue to dominate renewable energy, increasing by 3–5 times and 6–9 times by 2040, respectively.^5,6^ The large-scale energy transition toward wind and solar PV energy will inevitably require the construction of relevant infrastructure, thus raising concerns about their associated mineral material requirements.^7−17^

A growing body of research is looking at material demands for future wind and solar PV energy sectors. Generally, the generation and access to these renewable powers require two crucial parts to work together to realize a grid-connected renewable energy system. One type is electricity generation systems, which use wind turbines or solar PV panels and other auxiliary facilities (e.g., foundations and towers) to convert wind or solar radiation into electricity. The other is their associated electrical grid systems, which are the indispensable bridge connecting the power supply and demand sides by collecting the electricity generated by each generator and delivering electricity from renewable energy plants to the existing regional or national grids. Nevertheless, the majority of previous research evaluating material requirements has focused on electricity generation systems of wind and solar PV sectors, particularly wind turbines and solar PV panels,^18−26^ while little research has been done on their associated electrical grid systems. For instance, some researchers calculated future material requirements for the electricity generation of global offshore wind farms. Still, meanwhile, they pointed out that their study excluded equipment for associated electricity transmission due to the complexities of transmission.^27^

Moreover, while there have been some similar studies involving electricity transmission grids, these studies have been limited by different research aims and scopes to answer the question of how many mineral resources are needed for electrical grid systems linked to future global wind and solar PV. First, previous studies on metal requirements have tended to estimate in an aggregated manner, taking the global or regional grid network as a whole and packaging all types of electricity networks together, including all transmission and distribution grids as well as those that might be connected to fossil energy.^28,29^ The heterogeneity of electrical grid infrastructures associated with renewable development, such as the differences in power cables used for offshore and onshore wind, and their inter-field and export transmission lines have not been captured.^30−33^ Second, the impact of the evolution of renewable energy projects on their electrical grid systems, such as the distance to the main network connection point and the scaling up of individual renewable projects, has not been considered. Third, the recycling potential of mineral resources used in electrical grids has been missing. Finally, although some other studies have tried to cover the relevant electrical grid systems for renewables,^34,35^ for example, the life cycle impacts of transmission grid extensions arising from renewables in the European region have been examined,^35^ these studies either only provide environmental impact assessments of electricity grids for a specific technology (offshore) or are limited to region-specific grids.

Here, we develop a material demand model for electrical grid systems that integrates typical transmission grid engineering design related to wind and solar PV power sectors with dynamic material flow analysis (MFA). The global metal demand for electrical grid systems associated with these two dominant renewable energy technologies, as well as the potential for secondary metal supply by 2050, is quantified. We investigate the typical engineering parameters as well as the possible development trends of these electrical grids and differentiate the power cables, transformers, and substations in different energy technologies. Based on this, we then estimate the metal demands needed to satisfy the development of electrical grid networks directly associated with wind and solar PV in three International Energy Agency (IEA) energy scenarios. We include three bulk metals (copper, aluminum, and steel), which are the main minerals used in electrical grid systems. Such a detailed and in-depth analysis of the metal requirement for associated electrical grids with wind and solar PV allows a better understanding of the nexus of electricity networks, renewable energy, and mineral resources.

## 2 Methodology

### 2.1 Model Overview and Framework

**Figure 1:**
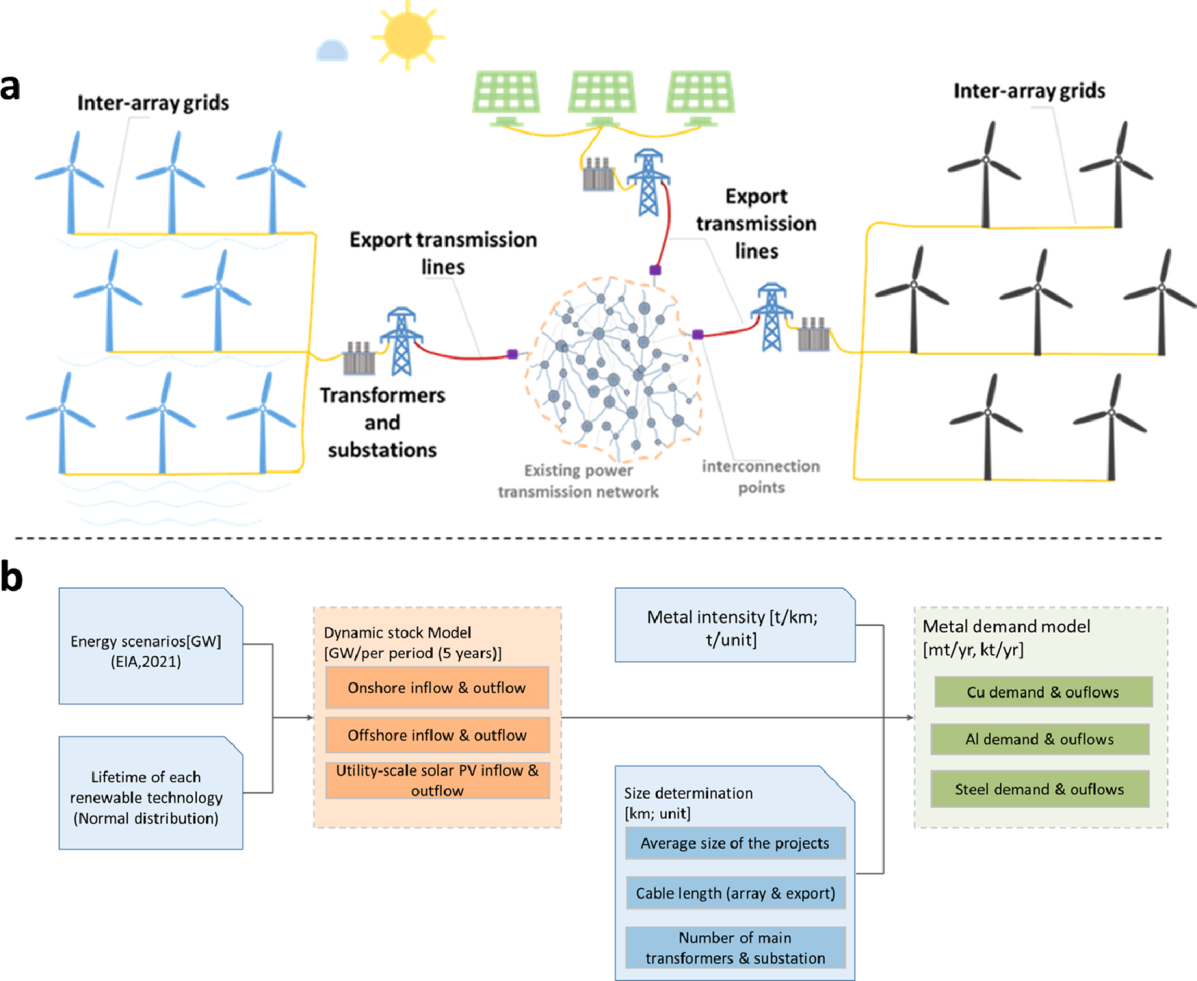
(a) Schematic diagram of the electrical grid systems covered in this study and (b) model framework for the metal requirement of electrical grid associated with wind power and utility-scale solar PV power.

The system definition and modeling framework for converting energy scenarios into metal demand for electrical grid systems are summarized in Figure 1. This study estimates the metal demands for building the electrical grid systems of the power plants for two major types of renewable energy technologies: wind power (including onshore and offshore wind) and utility-scale solar PV. Solar PV and wind have grown dramatically globally, accounting for more than half of total installed renewable capacity in 2020.^36^ These two are expected to continue dominating the renewable market, accounting for around 78–81% of all renewables and 50–71% of the total electricity mix in 2050.^37^ Therefore, the metals required for the electrical grid of these two renewables can essentially represent the overall demand trend for grid materials directly related to renewable energy technologies. Besides, data on grid systems for other renewable technologies are limited. Taking these considerations into account, we select these two types of renewable energy technologies as representatives. It should be noted here that the common forms of solar PV generation are distributed PV and utility-scale PV.^38^ It is generally believed that utility-scale PV will dominate electricity generation because of its favorable economies of scale, outweighing the savings in transmission costs brought by distributed PV.^5^ Additionally, distributed PV is generally locally consumed and is not connected to the transmission grid. Considering the above-mentioned and data availability, this study only includes utility-scale solar PV technology.

The electrical grids of these two renewables mainly consist of inter-array grids, export transmission lines, as well as transformers and substations. Inter-array grids collect power from individual wind turbines or solar PV panels and transmit it to the electricity collector platform, while export transmission lines transmit the power from power plants to existing main grids at interconnection points. Various power cables form the backbone of these transmission lines, which are also the focus of the study. Transformers and substations are built within these grid systems to step up or down the voltage to a level suitable for efficiently transmitting energy. Here, we only consider the “power (main) transformers” installed by transmission system operators,^39^ and other distribution transformers that power generation equipment manufacturers usually provide (e.g., wind turbines and transformers) are not included.^39^ Three bulk metals, copper, aluminum, and steel, are incorporated in our model. These three are the most widely used metal materials and are strategic to the production of most technologies, given the expected metal-intensive low-carbon energy and electrification.^40,41^ The demand for these three materials has been growing rapidly in recent years. Such rapid growth may cause future supply problems and environmental issues. For example, the growth of copper demand has been higher than the growth of its secondary resource due to the growing demand for primary copper.^42^ Meanwhile, the production of these materials is already energy-intensive and is a significant contributor to global greenhouse gas (GHG) emissions, and the decline in ore grades would further result in higher energy consumption and emissions for the same amount of metal extraction. Further, copper, aluminum, and steel are the main metals contained in grid-relevant electrical components. Aluminum and copper are the two main conductor materials in power cables, while steel is the protective and supporting structural material in power cables and transformers and substations. These three metals are able to represent the metal demands for electrical grids. Finally, it is important to note that not only does the construction of these two types of renewable power plants lead to the expansion of power transmission infrastructure, but other factors such as upgrading the existing grids, replacing aging transmission lines, electricity trading across borders and continents, and grid connection of other types of power plants, can also lead to the expansion of power grid network. This is, however, not included in the scope of our current research. This study only considers electrical grid systems that are “directly” related to the wind and solar PV energy projects, that is, infield and external transmission systems that are built together with the power projects, which means these unless otherwise indicated, the electrical grid systems mentioned in this article only refer to this type.

A prospective dynamic MFA method is used to simulate the relevant metal flows. Combined with the MFA, a model is developed based on typical engineering design models of electrical grid technologies to translate future installed wind and PV power capacities into metal requirements of electricity grid facilities, respectively. Relevant engineering design parameters, such as project size, distance to the main transmission grid, and electrical equipment selection, are considered, as well as their possible future development trends.

Metal requirements are quantified in a stepwise procedure. First, the installed wind and utility-scale solar PV capacity per period are calculated. Then, the most representative design parameters and engineering data of the electrical grid system for wind and utility-scale solar PV projects are determined, as well as their future characteristics over time. The final step is to use the results of the first two steps and some other external parameters (metal intensities) in the metal demand model to calculate the corresponding metal flows (see Figure 1). All calculations are performed in 5-year time steps to capture the most significant features and eliminate minor disturbance fluctuation factors.

### 2.2 Energy Scenarios and the Dynamic Stock Model

Our estimates for wind and utility-scale solar power developments are based on the energy scenarios developed by IEA.^43^ Three main scenarios are used: the Stated Policies Scenario (STEPS), the Sustainable Development Scenario (SDS), and the Net-Zero Emissions (NZE) by 2050 Scenario.^5^ These three scenarios chart different energy technology pathways by considering different assumptions about multiple key parameters (GDP, population, energy market dynamics, etc.). Among these energy scenarios, the NZE sets the most ambitious goals of energy transition and GHG emission, while the SDS outlines moderately ambitious but realistic energy planning, and the STEPS is based on stated policies and much less ambitious (detailed descriptions can be found in the Supporting Information).

We use the SDS as the baseline scenario and the other two scenarios as comparisons to show the impact of different baseline assumptions on the metal demand for the transmission infrastructure. The future electricity capacities of wind and solar PV are extracted from these three background scenarios. Two modifications are made to align the scenario data with our research scope. All the capacity information for solar PV in the IEA’s scenarios is the sum of distributed PV and utility-scale PV. Therefore, according to the proportion reported by the IEA (60–80%) and DNVGL (67%).^44−46^ we set the proportion of installed capacity of utility-scale solar PV at 70%. Additionally, as these energy scenarios only provide their demand implications every 10 years, we interpolate the annual scenario data and then gather data of every 5 years.

To determine the annual inflow (newly installed) and outflow (decommissioned) of power capacity, we consider the electricity capacity data from scenarios as stocks and use a dynamic stock-driven MFA model.^47,48^ The relationship between the stocks, newly installed and decommissioned capacities can be expressed as a convolution:
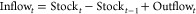
1
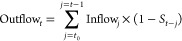
2where Inflow_*t*_ and Inflow_*j*_ are the newly installed capacities in year *t* and *j*, respectively. Stock_*t*_ and Stock_*t* – 1_ are the in-use capacities of wind and solar PV in year *t* and *t* – 1, respectively. Outflow_*t*_ is the decommissioned capacities in year *t*. *S*_*t* – *j*_ is the survival rate, representing the possibility of previously installed capacities that have not reached the end-of-life and survived after *t* – *j* years. The survival rate is determined based on the cumulative distribution function of the normal distribution. Generally, the expected lifetime of transmission power cable for national transmission and distribution grids is more than 40 years,^49^ and transformers are expected to last around 35 years.^50^ However, the electrical grid system currently studied only includes the grids within the scope of infield grids and export transmission lines for renewable power plants. Assuming once the plants are decommissioned, these auxiliary electrical grid facilities will also no longer be used. With this in mind, the lifetime of the electrical grid is assumed to be the same as that of these wind and solar PV power plants. So here the average lifetimes of electrical grid facilities for wind farms and PV plants are set to 20 and 25 years, respectively,^51−54^ and the standard deviation is set to 5 years.

### 2.3 Engineering Parameters of the Electrical Grids

The engineering parameters of wind and solar PV plant projects, such as the site selection, project scale, layout design of inter-array grids, export transmission line design, and other engineering parameters for individual projects, vary according to the technical type and specific requirements. For example, in terms of the project size, the average size of offshore wind farms over the years has far exceeded the average size of their onshore counterparts. The average size of offshore wind farms in 2015 reached 326 MW, far exceeding the average size of onshore wind farms (70 MW). There could be quite different design choices even for the same power plant projects. To capture possible and rational development features of future wind and solar PV projects, we check the relevant project technical reports and the literature,^55−77^ and estimate the most typical engineering designs of these projects at present and their future development trends. Full details can be found in Section 1.2 in the Supporting Information.

### 2.4 Metal Intensities of the Electrical Grids

The material composition and intensity of electrical equipment used in electrical grid systems for these two renewable technologies vary greatly. Taking the cable, which is the main component of the electrical grid as an example, the cable types used in power plants for wind and PV technologies are quite different. The first difference is the application scenarios of cables. Offshore wind farms require submarine cables, which are usually buried in the seabed. Submarine power cables are equipped with single or double amour (e.g., stainless steel wire armor) to protect cables from seawater corrosion and external impact, but this also results in a larger diameter and mass.^78^ Generally, underground cables are used for the infield-array grid system of onshore wind farms, while the export cable can either be underground or overhead. The underground cable is equipped with lead sheet and armoring, providing protection against moisture and mechanical injury. Overhead transmission cables use bare conductors and are placed high above the ground. The infield grid of solar PV plants requires both DC (direct current) and AC (alternate current) cables, which are designed to be UV and weather resistant.

The second typical difference is the rated voltage of the electrical grids. For example, offshore wind farms’ voltage levels of inter-array grids generally range from 20 to 66 kV,^61,79−84^ while the voltage levels of export transmission lines are typically between 100 and 320 kV.^85−87^ The difference in grid voltage leads to a difference in conductor cross-sectional area and conductor internal structure, which greatly influences the metal intensity.

The last significant difference is in the choice of the conductor. Copper and aluminum are the two main metals in cables. Copper is widely used in submarine cables and underground cables due to its excellent performance advantages, despite its higher price. However, aluminum is often used in overhead lines for its weight advantage and sometimes also used for submarine and underground cables.^32,88−90^ In addition, the metal contents of power transformers of onshore and offshore also differ. Offshore transformers, for example, tend to use more steel for support and protection.

Considering the above factors, we compile the typical metal contents or intensities of these power cables, transformers, and substations after reviewing the relevant literature, technical reports, and product manuals (see the SI). We assume that there are no revolutionary breakthroughs in transmission cable and substation technology in the future, and thus, their metal compositions and contents would be fixed in this study. The estimated results for metal intensities are shown in Table S2 in the Supporting Information.

## 3 Results

### 3.1 Energy Capacity Dynamics and Grid Length Requirements

**Figure 2:**
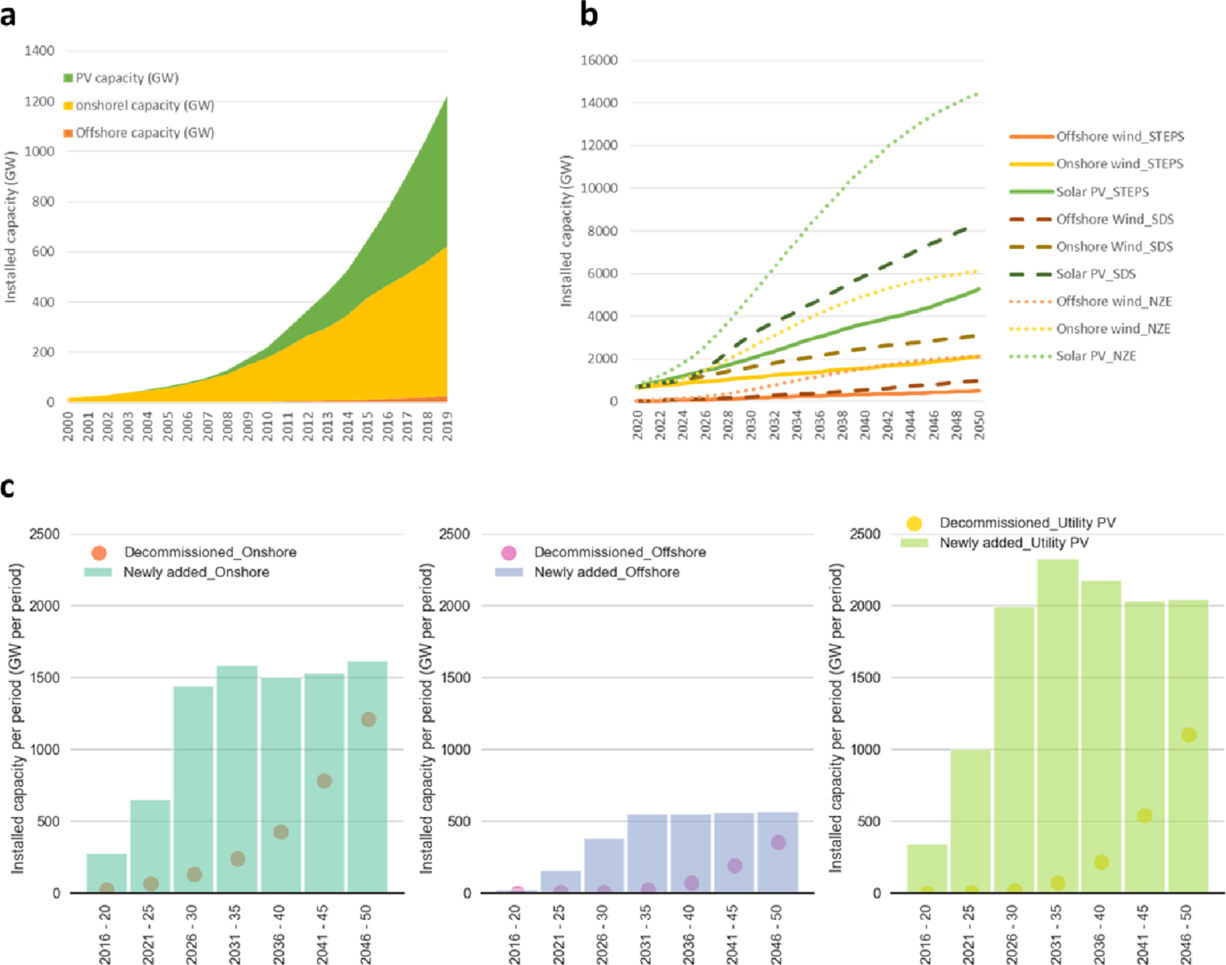
Generating capacities (stock capacities) and newly installed capacities per period for global renewable power systems. (a) Historical generating capacities of global utility-scale solar PV and wind power from 2000 to 2019. (b) Future possible global PV and wind power generating capacities toward 2050 under three IEA’s energy scenarios (STEPS, SDS, and NZE). (c) Newly installed capacities per period for utility-scale PV and wind (onshore and offshore) power under the NZE scenario.

Among the wind and utility-scale solar PV energy technologies, the installed capacity of the utility-scale PV remains the largest regardless of the energy scenario, followed by onshore wind power and offshore wind power. Furthermore, the generating capacity of the same renewable technology varies considerably in the scenarios, and this difference leads to different installed capacity additions in each period (Figure 2).

In the STEPS and SDS, the newly installed capacity per period for wind power, and utility-scale solar PV both present a continuous upward trend (Figure S2). However, the NZE sees a different development trajectory (Figure 2c). In the NZE, the newly installed capacity per period of both onshore and offshore wind power will show a significant growth trend between 2021 and 2030, subsequently, the newly installed capacity per period for onshore and offshore power will level off at 1550 GW and 550 GW, respectively. As for utility-scale solar PV power, its newly installed capacity per period will peak at around 2300 GW during the 2030–2035 period, and then gradually decline to around 2000 GW in 2046–2050. Additionally, because of historical accumulation and the surge in renewable energy in the coming decades, the decommissioned power capacity per period will also increase rapidly under all three scenarios, from a tiny amount in the 2021–2025 period to tens or even hundreds of times that in 2046–2050.

**Figure 3:**
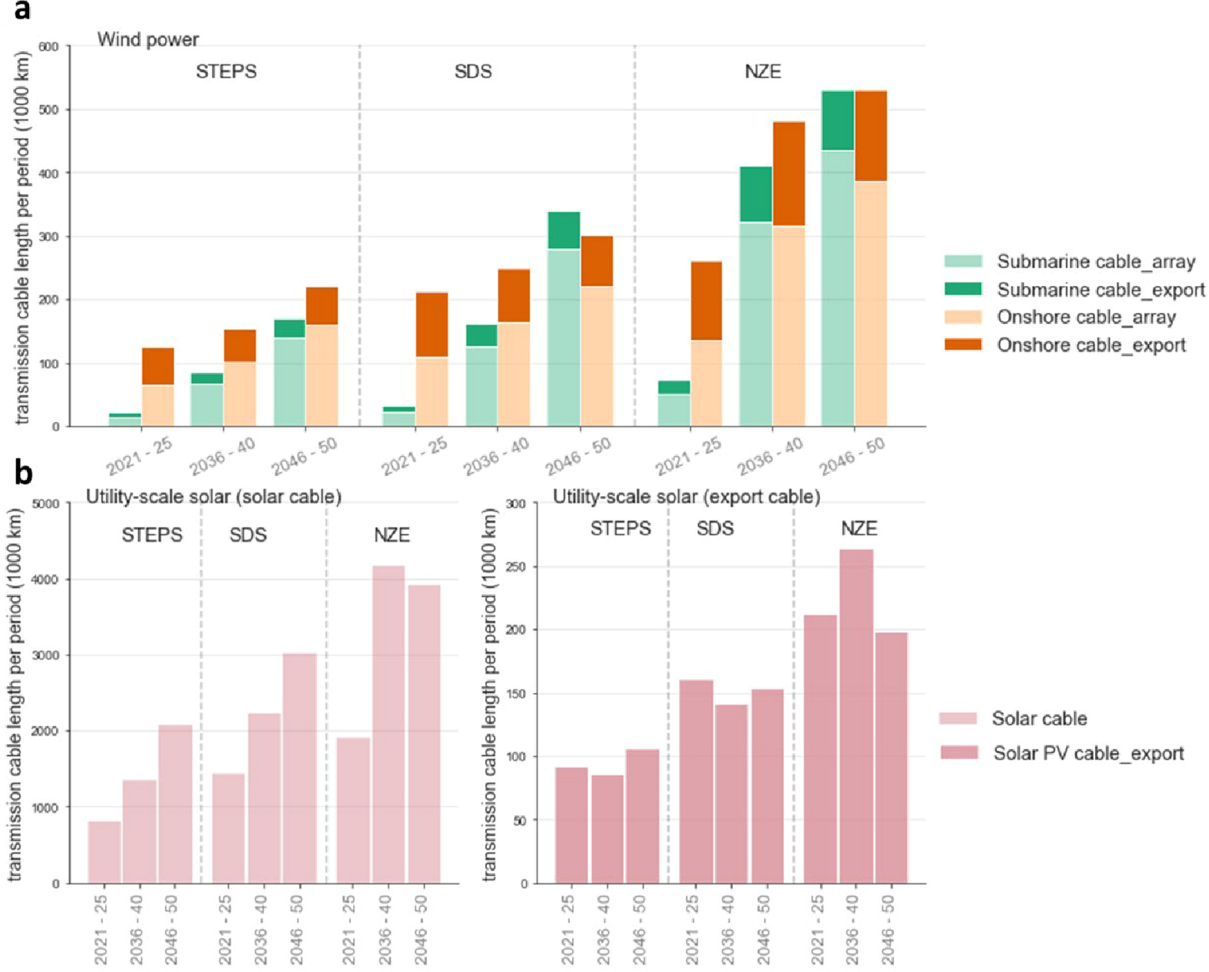
Grid length requirements resulting from renewable power projects under the STEPS, SDS, and NZE. (a) Inter-array and export submarine cable length for offshore wind projects; array and export onshore cable length for onshore wind projects. (b) (Array) solar cable and export cable length for utility-scale solar PV projects. Note: the vertical scales are different.

Figure 3 shows the grid length requirement accompanied by wind and utility-scale power by 2050. From the perspective of the long-term energy scenario setting, the more ambitious the installed capacity target of wind and PV power, the greater the total length of the power transmission cable. The total required cable length for both wind and PV technologies increases in order in all scenarios. From the perspective of technology breakdown, the total cable length required to build utility-scale solar PV projects is the longest, followed by onshore wind and offshore wind. This is partly because the expected installed capacity of PV is higher than that of wind in all scenarios, and partly because of the cable length coefficient used in current research. The data on the cable length coefficient of in-field solar cable for PV projects are very limited. Different length coefficients can lead to different length calculation results, which is why we emphasize the importance of statistical data and knowledge of the related transmission grids. Another feature is that although the overall installed capacity of offshore wind is smaller than its onshore counterpart, its cable demand is significant. Two development trends would cause this: one is the greater distance to shore for future offshore wind projects, and the other is their larger project sizes that could lead to more complex inter-array grids, which would exponentially increase the total length of array cable demand, based on our existing empirical formula S1 (see detailed information in the SI).

### 3.2 Metal Demand for the Electrical Grid Systems

**Figure 4:**
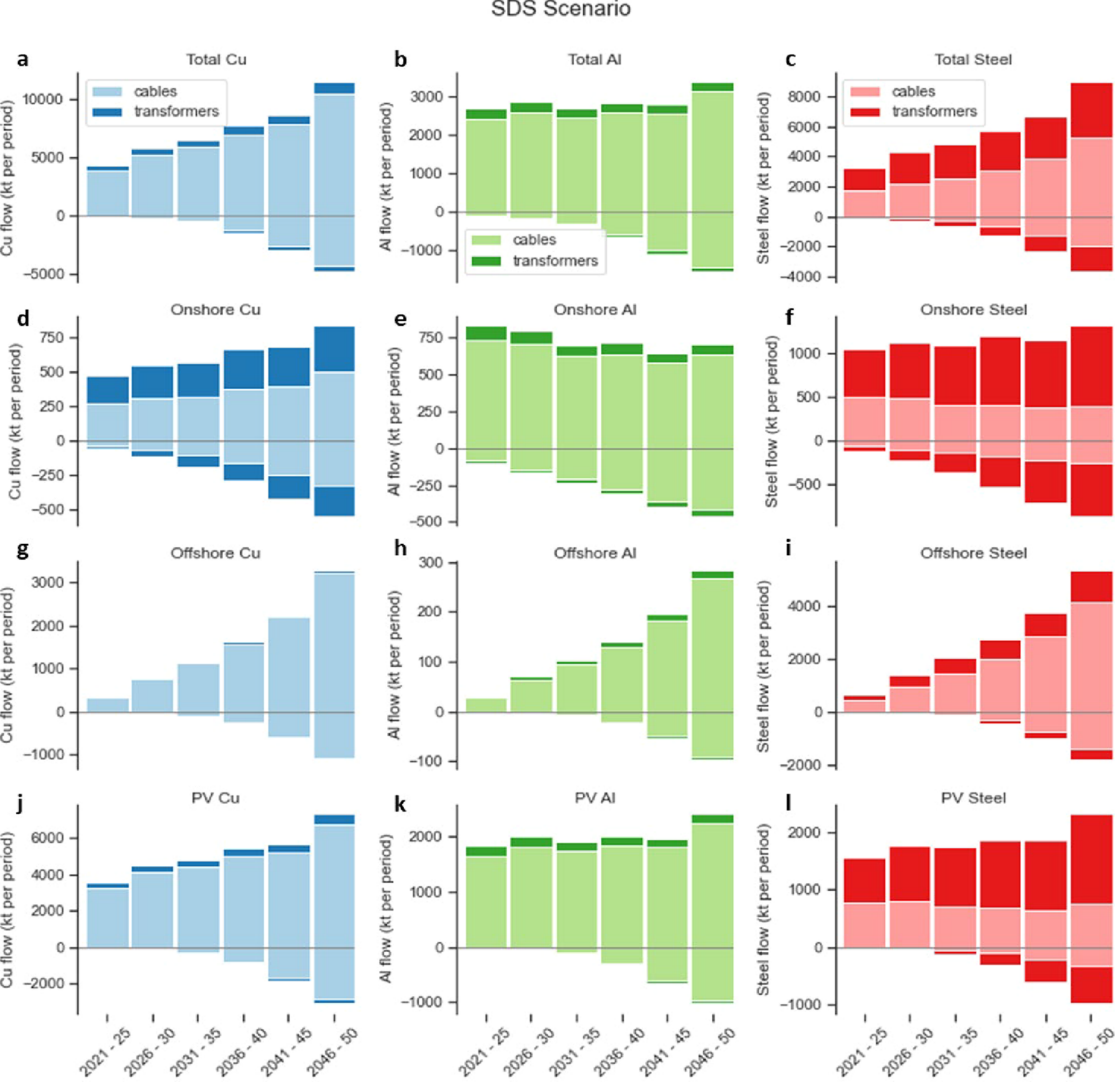
Metal demands (inflows) and corresponding decommissioned metal (outflows) for each period of newly built electrical grids associated with wind and utility-scale solar PV projects toward 2050 in the SDS scenario by technology. Total demands and decommissioned outflows of electrical grids for (a) copper, (b) aluminum, and (c) steel. The metal inflows and outflows of electrical grids result from (d–f) onshore wind projects, (g–i) offshore wind projects, and (j–l) utility-scale solar PV projects by 2050. Here, light shades represent metals contained in cables, dark shades represent metals contained in main transformers and other electrical equipment. Note: positive values on the *y* axis represent the metal inflows, and negative values represent the metal outflows. The vertical scales are different. Metal demands in the other two scenarios can be found in Figures S4 and S5.

**Figure 5:**
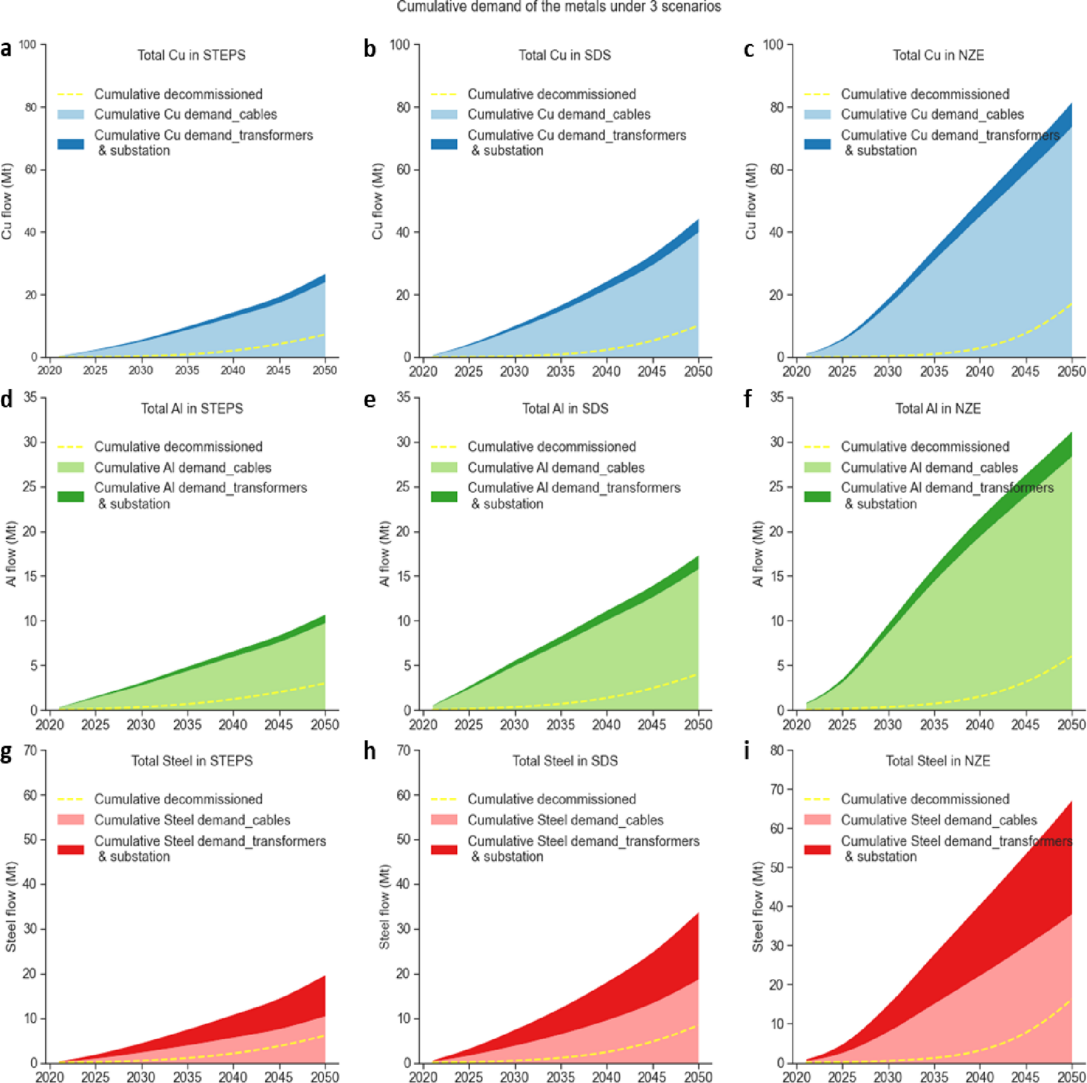
Cumulative metal demand and EOL outflow for the dynamics of electrical grids accompanying wind and utility-scale solar projects over time by 2050. (a–c) Cumulative copper demand and decommissioning, (d–f) cumulative aluminum demand and decommissioning, and (g–i) cumulative steel demand and decommissioning under the STEPS, SDS, and NZE scenarios. Here, we distinguish between metals contained in cables (light shade) and transformers and substations (dark shade).

Our results indicate that in the SDS, from the 2021–2025 period to the end of the modeling period, the copper demand per period for electrical grids of wind and solar technologies is going to grow from about 4.3 to 11.4 Mt; the aluminum demand in each period is relatively stable, increasing slightly from 2.7 to 3.4 Mt; and the steel demand per period will increase from 3.2 to 9.0 Mt (Figure 4). Regarding the cumulative demand, copper leads the way, followed by steel and aluminum. A cumulative total of 44 Mt of copper, 33 Mt of steel, and 17 Mt of aluminum would be required between 2021 and 2050, respectively (Figure 5). Among them, the use of cables accounts for around 90% of the cumulative demand for copper (97%) and aluminum (87%), while only 55% of that is for steel. This suggests that the vast majority of copper and aluminum contained in transmission lines will be locked into cable parts of the electrical systems, while almost half of the steel will be locked into the cables and the other half in the transformers and substations by 2050.

We compare our estimates with the current global production of three metals and relevant literature on future material demand to provide additional context for the results. The annual aluminum and steel demands for electrical grid systems directly associated with wind and utility-scale solar PV over the 2046–2050 period are small compared to their global productions in 2020^91,92^ (1.0 and 0.1%, respectively), while annual copper demand during 2046–2050 for electrical grids would account for a relatively large share, about 11.4% of global copper production in 2020.^93^ Furthermore, according to some estimates,^94^ the global energy transition requirement for copper and aluminum would range from 9 to 15 Mt, and 25 to 42 Mt, respectively, in 2050 under IEA’s energy scenarios. Comparing these figures with our findings shows that in 2050, the copper required to build the relevant grid systems would account for 9–16% of overall copper demand for the energy transition, while aluminum would account for only about 2% of the total.

Comparing our results with metal demand results from other energy scenarios^95^ shows that electrical grid systems would require around 12∼18% of cumulative copper required for future home appliances, cars and energy technology. Looking at individual technology, the cumulative aluminum demand for building the electrical grids of solar PV would account for 12% of the demand for developing solar technology itself (103 Mt).^96^ Furthermore, our results are compared with other categories of metal demand. In 2050, the global power system is expected to require 3–4.4 kt of neodymium and 3.7–14 of cobalt, respectively.^30^ Using our estimated aluminum demand as an example, electrical grid systems associated with wind and solar PV technologies would require hundreds of times more aluminum than neodymium and tens of times more cobalt, respectively.

From our estimates, although no major supply issues are expected for grid-related metal demand, building the electrical grid systems related to wind and solar PV may play an increasingly important role in future material requirements to some extent. In addition, since our model only considers the grid expansion directly related to the two types of renewable energy, it does not include the renovation and upgrading of the main grid indirectly related to these two energy technologies, and the grid expansion caused by other types of renewable energy technologies, so the actual metal demand for power transmission grids will be higher. Therefore, it is important to continuously monitor the future supply, consumption and criticality changes of metals used in electrical grid systems associated with wind and solar PV power technologies.

There are several interesting findings when looking into the metal demand for individual renewable technology transmission grids. First, among the renewable energy technologies involved, the solar PV-related electrical grid has the largest metal cumulative demands (see Figures S6–S8). Second, the electrical grid built for offshore wind power requires more steel than that for onshore wind and solar PV power. This can be explained by the high steel content of offshore electricity transmission components. Third, the offshore wind electrical grid will require more copper, while the onshore wind grid requires more aluminum because more overhead (aluminum) cables will be used on land.

We compare metal demand per period (see Figure S3) and their cumulative demand (Figure 5) for transmission grids under three scenarios to assess the impact of climate and energy policy on the metal demand for electrical grids. The results demonstrate that as the expected installed capacity of renewable power in the STEPS, SDS, and NZE scenarios increase in sequence, the metal demands for their electrical grid systems also grow accordingly. The NZE scenario has the most massive push for clean electrification, which is based on the drastic development of wind and solar, enabling a ramping up of progress on the electrical grid system and consequently increasing the demand for their corresponding metals. This also validates the fact that the energy transition and climate policies are indeed metal-intensive, from the new perspective of the electrical transmission grid, except from the perspective of already widely discussed solar cells, wind turbines, and EVs.^31,95,97,98^

### 3.3 Potential for Secondary Metal Supply

The outflow of copper, aluminum, and steel contained in decommissioned electrical grid systems associated with wind and utility solar PV sees a continuous increase, growing to 4.8, 1.6, and 3.7 Mt in the period 2046–2050 in the SDS, respectively (Figure 4). Accumulatively, 10 Mt of copper, 4 Mt of aluminum, and 8.4 Mt of steel flow out of the electrical grid in 2021–2050 (Figure 5). By calculating the ratio of the metal outflow to the metal inflow in this period, we can understand the extent to which potential secondary metal resource supply in the electrical grid could relieve its metal mineral supply. The calculation results show that if all metal flows contained in the decommissioned electrical grid were recycled and reused, the cumulative outflows of copper, aluminum, and steel could avoid more than 20% of virgin metal demand in 2021–2050. In terms of time dynamics, the outflows are negligible before 2035. But after that, as the earlier built wind farms and solar PV farms gradually reach their end of life, the electrical grid facilities will be shut down as part of the whole project, even though these power transmission lines have a longer lifetime and have not yet reached their end of life. During the 2046–2050 period, the outflows of all three metals could supply over 40% of the demand if all these metals were fully recycled or reused. Moreover, the remaining metal demand gap might be filled by decommissioning materials from the renewable energy generation system and other types of renewable systems.

Furthermore, our results show that an ambitious energy transition scenario would increase the gap between metal inflows and outflows of the electrical grid. The difference between the cumulative metal inflow and outflow for copper gradually increases to 20, 33, and 65 Mt respectively. This is caused by the average 20–25 year delay between the commissioning and decommissioning of these renewable power projects and the fact that metal inflows are generally lower in the early years of all three scenarios than in the later years, which results in a lower build-up of electrical grid available for decommissioning at the end of the studied period.

Although the three bulk metals covered in this study, copper, aluminum, and steel are generally among the metal categories with the highest recycling rates, the picture changes when the scope is narrowed to grids used to support renewable energy projects, especially for those used for offshore wind projects and underground parts. For overhead transmission lines that are usually built as export lines for onshore wind and solar PV projects, every part, including bare conductors, is easy to dismantle, recycle, or reuse, while for transmission lines that are buried underground and under the sea, their decommissioning is much more complicated. Although agreements have been reached across the industry and legal entities for removing wind turbines and their foundations, the decommissioning of their power grids remains highly controversial due to environmental and economic cost considerations, and they are now commonly abandoned on the ocean floor.^99−101^ Out of similar considerations, underground power cables are also left under the ground.^102^ According to our estimates, the submarine and underground power cables determine a significant fraction of the total metal demand. For instance, under the SDS scenario, by 2050, the submarine cables directly related to offshore wind power projects will contain about 26 Mt of three bulk metals. Therefore, to effectively utilize these potential submarine and underground urban mines, it is urgent for the industry and governments to address the decommissioning issue of the submarine and underground power cables and establish standardized regulations and systems for the decommissioning management of electrical grids. But at the same time, stakeholders need to consider the potential rebound effects of the circular economy.^103^ Improving the recycling of related grid components alone may not guarantee the reduction of the production and demand of related metal materials and environmental improvement. This point also needs to be carefully considered and balanced in the formulation of relevant regulations.

### 3.4 Uncertainties and Sensitivity

Our model outputs are based on a set of assumptions on variables such as the lifetime distribution of power projects, the metal intensities, array cable length for individual projects, length coefficient of inter-array cable (solar PV) and distance to main grids. A sensitivity analysis is thus performed to understand where the major uncertainty may arise, as well as assessing the impact of modeling assumptions on the simulation outcomes. All alternative simulation processes are placed in the SDS scenario, and only copper is tested. The sensitivity analysis results confirm that our model and results are robust to all key variables (see the SI for details).

## 4 Discussion

This research estimates metal demands for building inter-array power grids and export power transmission lines for wind and utility-scale solar PV. The results show that about 90 Mt of copper, aluminum, and steel would be required between 2021 and 2050 in the SDS. In the NZE scenario, this figure would be around two times higher (180 Mt). In either scenario, copper has the largest share of demand among the three considered metals (SDS: 44 Mt, 49%; NZE: 82 Mt, 46%), while demand for aluminum is relatively diminutive (SDS: 17 Mt, 19%; NZE: 31 Mt, 17%). Regarding renewable energy technologies, offshore wind and utility-scale solar projects require more copper for their electrical grids, while onshore wind projects require more aluminum. This is understandable. First, the conductors of the inter-array grids and export transmission lines of the current offshore wind projects generally prefer copper conductors, taking into account the more demanding operating environment and the superior performance of copper. Second, copper conductors are also the preferred option due to the harsh requirements of cables for the PV infield grid. However, the market price of copper is about three times as high as aluminum in the last decade.^104,105^ Considering this economic cost, grid operators are trying to switch from copper conductors to aluminum conductors. Despite some technical drawbacks, this is possible as technology advances. If the share of aluminum conductors contained in cables rises from our original assumption of 16 to 30% in the SDS, the cumulative primary copper demand by 2050 would be reduced by 5 Mt, while that for aluminum would rise by 4 Mt. Moreover, the industry is discussing the widespread adoption of new conductor materials – advanced conductors with carbon and/or composite cores that could provide significant emission reductions and customer savings, and replace conventional metal conductors.^106^

Another sustainable strategy to alleviate the metal demand for building associated electrical grid systems is extending the lifetime of these renewable energy projects. This can be achieved through lifetime extension measures or the repowering of decommissioned renewable projects. As we mentioned before, although wind farms and utility-scale solar plants are generally designed to last 20–25 years, their transmission lines can last much longer, typically around 35–40 years.^49,50^ Some of the operational lifetimes of larger power electrical networks, such as existing national transmission or distribution transmission lines, have exceeded 70 years.^106^ Extending the lifetime of aged renewable projects and repowering decommissioned renewable projects have many benefits, including taking advantage of superior natural endowments at appropriate sites, as well as making full use of existing electrical grid systems and other infrastructures already in place. If the lifetime of both wind and utility-scale solar PV projects is conservatively assumed to be extended to 30 years in the SDS, the cumulative primary copper and aluminum demand for associated electrical grids would be reduced by 6 Mt (down by 11%) and 2 Mt (down by 12%), respectively, by 2050. In the future, the continuous development of lifetime extensions for wind and solar PV projects, as well as the reform of legislation and approval policies for repowering, would undoubtedly help reduce the demand for metals in the associated electrical grid systems.

Finally, it is important to recognize that the growth of wind and PVs and grid expansion are not simply cause and effect; virtually any increase in electricity supply requires additional grid infrastructure. Even if the real world does not expand into renewable energy supply as assumed in the IEA’s energy scenario, additional electrical grid infrastructure would still be required to support the growing demand for electricity.

In general, this study evaluate the metal demands for building future electrical grid systems directly linked with wind and utility-scale solar PV power. Such a detailed analysis enables a deeper understanding of the impact of wind and solar PV technologies on electrical grids and the required metal resources, as well as the development trends and circular potential of these electrical grid infrastructures. This study is just an initial attempt to explore the direct impact of renewable power technologies on future electrical grids and the metals contained therein, further research should cover more renewable technologies and their impacts on electrical grids, metal resources, and environmental aspects.

## Supporting Information Available

Methods, results, and sensitivity analysis (PDF)

## References

[ref1] Global cumulative installed solar PV capacity 2020. Statista. https://www.statista.com/statistics/280220/global-cumulative-installed-solar-pv-capacity/ (accessed August 23, 2022).

[ref2] Wabukala B. M.; Otim J.; Mubiinzi G.; Adaramola M. S. Assessing Wind Energy Development in Uganda: Opportunities and Challenges. Wind Eng. 2021, 45, 1714–1732. 10.1177/0309524X20985768.

[ref3] Vrontisi Z.; Fragkiadakis K.; Kannavou M.; Capros P. Energy System Transition and Macroeconomic Impacts of a European Decarbonization Action towards a below 2 °C Climate Stabilization. Clim. Change 2020, 162, 1857–1875. 10.1007/s10584-019-02440-7.

[ref4] Creutzig F.; Agoston P.; Goldschmidt J. C.; Luderer G.; Nemet G.; Pietzcker R. C. The Underestimated Potential of Solar Energy to Mitigate Climate Change. Nat. Energy 2017, 2, 17140 10.1038/nenergy.2017.140.

[ref5] https://www.iea.org/reports/world-energy-outlook-2020

[ref6] IEA. Net Zero by 2050: A Roadmap for the Global Energy Sector; Paris, 2021, 10.1787/c8328405-en.

[ref7] Tokimatsu K.; Hök M.; McLellan B.; Wachtmeister H.; Murakami S.; Yasuoka R.; Nishio M. Energy Modeling Approach to the Global Energy-Mineral Nexus: Exploring Metal Requirements and the Well-below 2 °C Target with 100 Percent Renewable Energy. Appl. Energy 2018, 225, 1158–1175. 10.1016/j.apenergy.2018.05.047.

[ref8] Wang P.; Chen L.-Y.; Ge J.-P.; Cai W.; Chen W.-Q. Incorporating Critical Material Cycles into Metal-Energy Nexus of China’s 2050 Renewable Transition. Appl. Energy 2019, 253, 113612 10.1016/j.apenergy.2019.113612.

[ref9] Moreau V.; Dos Reis P. C.; Vuille F. Enough Metals? Resource Constraints to Supply a Fully Renewable Energy System. Resources 2019, 8, 29 10.3390/resources8010029.

[ref10] Deng X.; Ge J. Global Wind Power Development Leads to High Demand for Neodymium Praseodymium (NdPr): A Scenario Analysis Based on Market and Technology Development from 2019 to 2040. J. Cleaner Prod. 2020, 277, 123299 10.1016/j.jclepro.2020.123299.

[ref11] Rabe W.; Kostka G.; Smith Stegen K. China’s Supply of Critical Raw Materials: Risks for Europe’s Solar and Wind Industries?. Energy Policy 2017, 101, 692–699. 10.1016/j.enpol.2016.09.019.

[ref12] Vidal O.; Goffé B.; Arndt N. Metals for a Low-Carbon Society. Nat. Geosci. 2013, 6, 894–896. 10.1038/ngeo1993.

[ref13] Watari T.; Nansai K.; Giurco D.; Nakajima K.; McLellan B.; Helbig C. Global Metal Use Targets in Line with Climate Goals. Environ. Sci. Technol. 2020, 54, 12476–12483. 10.1021/acs.est.0c02471. 32915547

[ref14] Beylot A.; Guyonnet D.; Muller S.; Vaxelaire S.; Villeneuve J. Mineral Raw Material Requirements and Associated Climate-Change Impacts of the French Energy Transition by 2050. J. Cleaner Prod. 2019, 208, 1198–1205. 10.1016/j.jclepro.2018.10.154.

[ref15] Lèbre É.; Owen J. R.; Corder G. D.; Kemp D.; Stringer M.; Valenta R. K. Source Risks As Constraints to Future Metal Supply. Environ. Sci. Technol. 2019, 53, 10571–10579. 10.1021/acs.est.9b02808. 31432668 PMC9936542

[ref16] Elshkaki A.; Graedel T. E.; Ciacci L.; Reck B. K. Resource Demand Scenarios for the Major Metals. Environ. Sci. Technol. 2018, 52, 2491–2497. 10.1021/acs.est.7b05154. 29380602

[ref17] Kleijn R.; van der Voet E.; Kramer G. J.; van Oers L.; van der Giesen C. Metal Requirements of Low-Carbon Power Generation. Energy 2011, 36, 5640–5648. 10.1016/j.energy.2011.07.003.

[ref18] Fishman T.; Graedel T. E. Impact of the Establishment of US Offshore Wind Power on Neodymium Flows. Nat. Sustainability 2019, 2, 332–338. 10.1038/s41893-019-0252-z.

[ref19] Cao Z.; O’Sullivan C.; Tan J.; Kalvig P.; Ciacci L.; Chen W.; Kim J.; Liu G. Resourcing the Fairytale Country with Wind Power: A Dynamic Material Flow Analysis. Environ. Sci. Technol. 2019, 53, 11313–11322. 10.1021/acs.est.9b03765. 31455077

[ref20] Yang J.; Zhang L.; Chang Y.; Hao Y.; Liu G.; Yan Q.; Zhao Y. Understanding the Material Efficiency of the Wind Power Sector in China: A Spatial-Temporal Assessment. Resour., Conserv. Recycl. 2020, 155, 104668 10.1016/j.resconrec.2019.104668.

[ref21] Kim J.; Guillaume B.; Chung J.; Hwang Y. Critical and Precious Materials Consumption and Requirement in Wind Energy System in the EU 27. Appl. Energy 2015, 139, 327–334. 10.1016/j.apenergy.2014.11.003.

[ref22] Samuel C.; Patricia A.D.; Beatrice P.; Claudiu P. Raw materials demand for wind and solar PV technologies in the transition towards a decarbonised energy system, 2020.

[ref23] Li J.; Peng K.; Wang P.; Zhang N.; Feng K.; Guan D.; Meng J.; Wei W.; Yang Q. Critical Rare-Earth Elements Mismatch Global Wind-Power Ambitions. One Earth 2020, 3, 116–125. 10.1016/j.oneear.2020.06.009.

[ref24] Lee J.; Bazilian M.; Sovacool B.; Hund K.; Jowitt S. M.; Nguyen T. P.; Månberger A.; Kah M.; Greene S.; Galeazzi C.; Awuah-Offei K.; Moats M.; Tilton J.; Kukoda S. Reviewing the Material and Metal Security of Low-Carbon Energy Transitions. Renew. Sustainable Energy Rev. 2020, 124, 109789 10.1016/j.rser.2020.109789.

[ref25] Valero A.; Valero A.; Calvo G.; Ortego A.; Ascaso S.; Palacios J.-L. Global Material Requirements for the Energy Transition. An Exergy Flow Analysis of Decarbonisation Pathways. Energy 2018, 159, 1175–1184. 10.1016/j.energy.2018.06.149.

[ref26] de Koning A.; Kleijn R.; Huppes G.; Sprecher B.; van Engelen G.; Tukker A. Metal Supply Constraints for a Low-Carbon Economy?. Resour., Conserv. Recycl. 2018, 129, 202–208. 10.1016/j.resconrec.2017.10.040.

[ref27] Li C.; Mogollón J. M.; Tukker A.; Dong J.; von Terzi D.; Zhang C.; Steubing B. Future Material Requirements for Global Sustainable Offshore Wind Energy Development. Renew. Sustainable Energy Rev. 2022, 164, 112603 10.1016/j.rser.2022.112603.

[ref28] Li F.; Ye Z.; Xiao X.; Xu J.; Liu G. Material Stocks and Flows of Power Infrastructure Development in China. Resour., Conserv. Recycl. 2020, 160, 104906 10.1016/j.resconrec.2020.104906.

[ref29] Kalt G.; Thunshirn P.; Wiedenhofer D.; Krausmann F.; Haas W.; Haberl H. Material Stocks in Global Electricity Infrastructures – An Empirical Analysis of the Power Sector’s Stock-Flow-Service Nexus. Resour., Conserv. Recycl. 2021, 173, 105723 10.1016/j.resconrec.2021.105723.

[ref30] Deetman S.; de Boer H. S.; Van Engelenburg M.; van der Voet E.; van Vuuren D. P. Projected material requirements for the global electricity infrastructure – generation, transmission and storage. Resour., Conserv. Recycl. 2021, 164, 105200 10.1016/j.resconrec.2020.105200.

[ref31] Tokimatsu K.; Wachtmeister H.; McLellan B.; Davidsson S.; Murakami S.; Hök M.; Yasuoka R.; Nishio M. Energy Modeling Approach to the Global Energy-Mineral Nexus: A First Look at Metal Requirements and the 2 °C Target. Appl. Energy 2017, 207, 494–509. 10.1016/j.apenergy.2017.05.151.

[ref32] IEA. The Role of Critical Minerals in Clean Energy Transitions – World Energy Outlook Special Report; International Energy Agency (IEA), 2020.

[ref33] Jorge R. S.; Hertwich E. G. Grid Infrastructure for Renewable Power in Europe: The Environmental Cost. Energy 2014, 69, 760–768. 10.1016/j.energy.2014.03.072.

[ref34] Arvesen A.; Christine; Birkeland; Hertwich E. G. The Importance of Ships and Spare Parts in LCAs of Offshore Wind Power. Environ. Sci. Technol. 2013, 47, 2948–2956. 10.1021/es304509r. 23409942

[ref35] Berrill P.; Arvesen A.; Scholz Y.; Gils H. C.; Hertwich E. G. Environmental Impacts of High Penetration Renewable Energy Scenarios for Europe. Environ. Res. Lett. 2016, 11, 014012 10.1088/1748-9326/11/1/014012.

[ref36] International Renewable Energy Agency. Renewable Capacity Statistics 2021; IRENA Abu Dhabi, 2021.

[ref37] International Renewable Energy Agency IRENA. Transforming the Energy System; International Renewable Energy Agency (IRENA), 2019.

[ref38] Lumby B. Utility-Scale Solar Photovoltaic Power Plants: A Project Developer’s Guide; The World Bank, 2015; Vol. 99396, pp 1 −216.

[ref39] Van Tichelen P.; Mudgal S. LOT 2: Distribution and Power Transformers Tasks 1–7. VITO and Bio Intelligence Service: Paris, France, 2011.

[ref40] Schipper B. W.; Lin H.-C.; Meloni M. A.; Wansleeben K.; Heijungs R.; van der Voet E. Estimating Global Copper Demand until 2100 with Regression and Stock Dynamics. Resour., Conserv. Recycl. 2018, 132, 28–36. 10.1016/j.resconrec.2018.01.004.

[ref41] Hatayama H.; Daigo I.; Matsuno Y.; Adachi Y. Outlook of the World Steel Cycle Based on the Stock and Flow Dynamics. Environ. Sci. Technol. 2010, 44, 6457–6463. 10.1021/es100044n. 20704247

[ref42] Elshkaki A.; Graedel T. E.; Ciacci L.; Reck B. K. Copper Demand, Supply, and Associated Energy Use to 2050. Global Environ. Change 2016, 39, 305–315. 10.1016/j.gloenvcha.2016.06.006.

[ref43] https://www.iea.org/reports/world-energy-outlook-2021

[ref44] Schmela M.; Beauvais A.; Chevillard N.; Guillén Paredes M.; Heisz M.; Rossi R. Global Market Outlook for Solar Power 2018–2022; SolarPower Europe, 2018; pp 1–81.

[ref45] Solar PV – Renewables 2020 – Analysis – IEA. https://www.iea.org/reports/renewables-2020/solar-pv (accessed October 20, 2021).

[ref46] Olson D.; Bakken B. E. Utility-scale solar PV: From big to biggest. https://www.dnvgl.com/feature/utility-scale-solar.html (accessed October 20, 2021).

[ref47] Wang F.; Huisman J.; Stevels A.; Baldé C. P. Enhancing E-Waste Estimates: Improving Data Quality by Multivariate Input–Output Analysis. Waste Manage. 2013, 33, 2397–2407. 10.1016/j.wasman.2013.07.005. 23899476

[ref48] Liu G.; Bangs C. E.; Müller D. B. Stock Dynamics and Emission Pathways of the Global Aluminium Cycle. Nat. Clim. Change 2012, 3, 338–342. 10.1038/nclimate1698.

[ref49] Joint Research Centre, Institute for Energy and Transport; Ardelean M.; Minnebo P. HVDC submarine power cables in the world: state-of-the-art knowledge, 2017.

[ref50] Behi B.; Arefi A.; Pezeshki H.; Shahnia F. Distribution Transformer Lifetime Analysis in the Presence of Demand Response and Rooftop PV Integration. In World Renewable Energy Congress (WREC); eprints.qut.edu.au, 2017; p 6.

[ref51] Ziegler L.; Gonzalez E.; Rubert T.; Smolka U.; Melero J. J. Lifetime Extension of Onshore Wind Turbines: A Review Covering Germany, Spain, Denmark, and the UK. Renew. Sustainable Energy Rev. 2018, 82, 1261–1271. 10.1016/j.rser.2017.09.100.

[ref52] Srivastava R.; Tiwari A. N.; Giri V. K. An Overview on Performance of PV Plants Commissioned at Different Places in the World. Energy Sustainable Dev. 2020, 54, 51–59. 10.1016/j.esd.2019.10.004.

[ref53] Pakenham B.; Ermakova A.; Mehmanparast A. A Review of Life Extension Strategies for Offshore Wind Farms Using Techno-Economic Assessments. Energies 2021, 14, 1936 10.3390/en14071936.

[ref54] Bouty C.; Schafhirt S.; Ziegler L.; Muskulus M. Lifetime Extension for Large Offshore Wind Farms: Is It Enough to Reassess Fatigue for Selected Design Positions?. Energy Procedia 2017, 137, 523–530. 10.1016/j.egypro.2017.10.381.

[ref55] European Regional Development Fund. Future Energy Industry Trends. https://northsearegion.eu/northsee/e-energy/future-energy-industry-trends/ (accessed October 18, 2021).

[ref56] Díaz H.; Guedes Soares C. Review of the Current Status, Technology and Future Trends of Offshore Wind Farms. Ocean Eng. 2020, 209, 107381 10.1016/j.oceaneng.2020.107381.

[ref57] Enevoldsen P.; Valentine S. V. Do Onshore and Offshore Wind Farm Development Patterns Differ?. Energy Sustainable Dev. 2016, 35, 41–51. 10.1016/j.esd.2016.10.002.

[ref58] IEA. Average awarded project size in utility-scale solar PV, Europe and emerging markets, 2013–2017. https://www.iea.org/data-and-statistics/charts/average-awarded-project-size-in-utility-scale-solar-pv-europe-and-emerging-markets-2013-2017 (assessed October 2, 2021).

[ref59] Miller A. Economics of utility-scale solar in Aotearoa New Zealand, 2020.

[ref60] Musial W. D.; Beiter P. C.; Spitsen P.; Nunemaker J.; Gevorgian V. 2018 Offshore Wind Technologies Market Report; National Renewable Energy Lab. (NREL): Golden, CO (United States), 2019.

[ref61] Ramírez L.; Fraile D.; Brindley G. Offshore Wind in Europe: Key Trends and Statistics 2019, 2020.

[ref62] Beiter P. C.; Tian T.; Nunemaker J.; Musial W. D.; Lantz E. J.; Gevorgian V.; Spitsen P. 2017 Offshore Wind Technologies Market Update; National Renewable Energy Lab. (NREL): Golden, CO (United States), 2018.

[ref63] Garrett P.; Ronde K. Life Cycle Assessment of Electricity Production from an Onshore V126–3.3 MW Wind Plant; Vestas Wind Systems A/S, 2014.

[ref64] Garrett P.; Ronde K. Life Cycle Assessment of Electricity Production from an V126–3.45 Onshore Wind Plant Vestas; Vestas Wind Systems A/S, 2017.

[ref65] Mondol J.; Jacob G. Commercial Scale Solar Power Generation (5 MW to 50 MW) and Its Connection to Distribution Power Network in the United Kingdom. J. Solar Energy Res. Updates 2018, 5, 25–38. 10.15377/2410-2199.2018.05.4.

[ref66] Sward J. A.; Siff J.; Gu J.; Max Zhang K. Strategic Planning for Utility-Scale Solar Photovoltaic Development – Historical Peak Events Revisited. Appl. Energy 2019, 250, 1292–1301. 10.1016/j.apenergy.2019.04.178.

[ref67] Fischetti M.; Pisinger D. Optimal Wind Farm Cable Routing: Modeling Branches and Offshore Transformer Modules. Networks 2018, 72, 42 10.1002/net.21804.

[ref68] El Mokhi C.; Addaim A. Optimization of Wind Turbine Interconnections in an Offshore Wind Farm Using Metaheuristic Algorithms. Sustainability 2020, 12, 5761 10.3390/su12145761.

[ref69] Pillai A. C.; Chick J.; Johanning L.; Khorasanchi M.; de Laleu V. Offshore Wind Farm Electrical Cable Layout Optimization. Eng. Optim. 2015, 47, 1689–1708. 10.1080/0305215X.2014.992892.

[ref70] Kaiser M. J.; Snyder B. Offshore Wind Energy Installation and Decommissioning Cost Estimation in the Us Outer Continental Shelf; US Dept. of the Interior, Bureau of Ocean Energy Management, Regulation and Enforcement: Herndon, VA TA&R, 2010; Vol. 648.

[ref71] Schachner J. Power Connections for Offshore Wind Farms; na, 2004.

[ref72] Evans S. The biggest solar power plants in the world. https://www.power-technology.com/features/the-worlds-biggest-solar-power-plants/ (accessed October 20, 2021).

[ref73] White paper on solar DC cables. https://renewablewatch.in/2018/07/09/white-paper-solar-dc-cables/ (accessed October 20, 2021).

[ref74] Satpathy R. K.; Pamuru V. Solar PV Power: Design, Manufacturing and Applications from Sand to Systems; Academic Press, 2020.

[ref75] Borup U.; Grau H.; Lave B. String Inverters for PV Power Plants, 2009.

[ref76] Deutsches Windenergie-Institut; Tech-wise A/S; DM Energy. Wind Turbine Grid Connection and Interaction, 2001.

[ref77] Yuan J. Analysis of Wind Farm’s Connection modes, Grid Connection and Operation Modes; Ph.D. Dissertation; Shanghai Jiao Tong University: Shanghai, China, 2012 (in Chinese).

[ref78] Zhang H.; Zhang J.; Duan L.; Xie S.; Xue J. Application Status of XLPE Insulated Submarine Cable Used in Offshore Wind Farm in China. J. Eng. 2017, 2017, 702–707. 10.1049/joe.2017.0421.

[ref79] Baring-Gould I. Offshore Wind Plant Electrical Systems, 2014.

[ref80] DNV KEMA Renewables, Inc. Appendix D Substation and Cable Route Design Report; DNV KEMA Renewables, Inc., 2014.

[ref81] Hans de Boer A.; van der H. Inventory Offshore Wind Test Sites Demand & Supply in the Netherlands; BLIX Consultancy, 2016.

[ref82] 113799-UKBR-R02, Rev. 2; TenneT

[ref83] Sharples M. Offshore Electrical Cable Burial for Wind Farms: State of the Art, Standards and Guidance & Acceptable Burial Depths, Separation Distances and Sand Wave Effect; Risk & Technology Consulting Inc.: Offshore, 2011.

[ref84] Llc V. W. Draft Construction and Operations Plan Volume I – Vineyard Wind Project, 2020.

[ref85] Ankit Gupta A. S. B. Offshore Wind Cable Market size to exceed $3 billion by 2026. https://www.gminsights.com/pressrelease/offshore-wind-cable-market (accessed November 11, 2021).

[ref86] https://www.nexans.com/en/dam/jcr:717293e8-cb52-4dba-81f3-fd5370159a7b/Nexans%20Offshore%20Wind%20Farm%20WEB.pdf

[ref87] Weerheim R. Development of Dynamic Power Cables for Commercial Floating Wind Farms; Literature assignment, 2018.

[ref88] Boone W.; Sonderen C. Copper in Comparison with Aluminium as Common Material in Conductors of Lv and Mv Cables. In Proceedings of 23rd International Conference on Electricity Distribution, Lyon, France, 2015; pp 26–25.

[ref89] Mueller-Schuetze S.; Ottersberg H.; Suhr C.; Krusche I.; Seekabelwerke N. Development of Submarine MV-AC Power Cable with Aluminum Conductor. 9th International Conference on Insulated Power Cables, 2015.

[ref90] Worzyk T.; Långström S. Use of Aluminum Conductors in Submarine Power Cables. In 9th International Conference on Insulated Power Cables, Versailles, Technical Paper, Versailles, France, 2015.

[ref91] Primary aluminium production. International Aluminium Institute. https://international-aluminium.org/statistics/primary-aluminium-production/ (accessed February 13, 2022).

[ref92] Global crude steel output decreases by 0.9% in 2020. worldsteel.org. https://worldsteel.org/media-centre/press-releases/2021/global-crude-steel-output-decreases-by-0-9-in-2020/ (accessed February 13, 2022).

[ref93] International Copper Study Group. The World Copper Factbook 2021; International Copper Study Group, 2021.

[ref94] Gregoir L.; Acker V. K. Metals for Clean Energy: Pathways to Solving Europe’s Raw Materials Challenge; KU Leuven, 2022. https://eurometaux.eu/media/20ad5yza/2022-policymaker-summary-report-final.pdf

[ref95] Deetman S.; Pauliuk S.; van Vuuren D. P.; van der Voet E.; Tukker A. Scenarios for Demand Growth of Metals in Electricity Generation Technologies, Cars, and Electronic Appliances. Environ. Sci. Technol. 2018, 52, 4950–4959. 10.1021/acs.est.7b05549. 29533657 PMC5906757

[ref96] Hund K.; La Porta D.; Fabregas T. P.; Laing T.; Drexhage J. Minerals for Climate Action: The Mineral Intensity of the Clean Energy Transition; World Bank, 2020; Vol. 73.

[ref97] de Koning A.; Kleijn R.; Engelen G.; Huppes G. Resource constraints in successful climate policy: Key constraints and bottlenecks, and some solutions. CECILIA2050 WP4 Deliverable 4.3; Institute of Environmental Sciences (CML), Leiden University, 2015.

[ref98] Habib K.; Hansdóttir S. T.; Habib H. Critical Metals for Electromobility: Global Demand Scenarios for Passenger Vehicles, 2015–2050. Resour., Conserv. Recycl. 2020, 154, 104603 10.1016/j.resconrec.2019.104603.

[ref99] Al-Sallami O. Cables decommissioning in offshore wind farms: environmental and economic perspective; Dissertation; Uppsala University, 2021. http://www.diva-portal.org/smash/get/diva2:1578274/FULLTEXT01.pdf

[ref100] Topham E.; McMillan D. Sustainable Decommissioning of an Offshore Wind Farm. Renew. Energy 2017, 102, 470–480. 10.1016/j.renene.2016.10.066.

[ref101] Hamburg Institute of International Economics. Market Analysis Decommission Tools 2019, 2020.

[ref102] Krook J.; Svensson N.; Wallsten B. Urban Infrastructure Mines: On the Economic and Environmental Motives of Cable Recovery from Subsurface Power Grids. J. Cleaner Prod. 2015, 104, 353–363. 10.1016/j.jclepro.2015.05.071.

[ref103] Zink T.; Geyer R. Circular Economy Rebound. J. Ind. Ecol. 2017, 21, 593–602. 10.1111/jiec.12545.

[ref104] Copper annual market price. Statista. https://www.statista.com/statistics/533292/average-price-of-copper/ (accessed April 20, 2022).

[ref105] Average prices for aluminum worldwide from 2014 to 2025. Statista. https://www.statista.com/statistics/675845/average-prices-aluminum-worldwide/ (accessed April 20, 2022).

[ref106] Jay Caspary J. S. Advanced Conductors On Existing Transmission Corridors To Accelerate Low Cost Decarbonization; Grid Strategies LLC, 2022.

