# Supplementary material for: Metal Requirements for Building Electrical Grid Systems of Global Wind Power and Utility-Scale Solar Photovoltaic until 2050
Source: Environ Sci Technol. 2022 Dec 29;57(2):1080–91. doi: 10.1021/acs.est.2c06496 (PMC9850911; doi:10.1021/acs.est.2c06496)
Supplement: Supplementary file 1 — es2c06496_si_001.pdf [file es2c06496_si_001.pdf]

Supplementary Information

Metal requirements for building electrical grid systems of global wind power and utility-scale solar PV until 2050

Zhenyang Chen[1], Rene Kleijn[1], Hai Xiang Lin[1, 2]*

[1.] Institute of Environmental Sciences (CML), Leiden University, 2333 CC Leiden, The Netherlands

[2.] Delft Institute of Applied Mathematics, Delft University of Technology, 2628 CD Delft, The Netherlands

*Corresponding author: E-mail: z.chen@cml.leidenuniv.nl

Pages: 60

Figures: 11

Tables: 23

1. Methods and data sources

1.1 The characteristics of IEA's energy scenarios

To explore global future energy pathways that meet the Paris climate goals and realize the transition to a low carbon economy, the IEA annually updates how the global energy system would develop over the next few decades. Multiple long-term scenarios have been explored by the agency due to future uncertainty. Three main scenarios are used in our research. The main features of the three IEA's energy scenarios are described as follows.

- The Stated Policies Scenario (STEPS) considers only those policies and commitments regarding energy to the environment that have been formally adopted or announced by governments, and it represents the least ambitious rate of transition to renewable energy. The consequences of these policies fall far short of the United Nations Sustainable Development Goals (SDGs). In this scenario, by 2040 the share of renewable capacity in total electricity capacity will rise to 58% (7738 GW), of which solar PV and wind will account for 47 (3655 GW) and 24% (1914 GW) of renewable energy respectively, and other renewables are hydro, bioenergy, geothermal, etc[1].
- The Sustainable Development Scenario (SDS) outlines an ambitious, but realistic and desirable energy planning by transforming the energy market to achieve the objectives of the Paris Agreement, including increasing the share of renewable energy and efficiencies, which holds the temperature rise below 2 °C with a 50% probability by late 21st century. The SDS assumes that renewable capacity will expand to 71% (11764 GW) of total electricity capacity by 2040, and rely much more on solar PV and wind, accounting for 50% (5891 GW) and 26% (3058GW) of total renewable capacity respectively[1].

- The Net-Zero Emissions by 2050 Scenario (NZE) extends the SDS analysis and is much more ambitious, which provides a 50% chance of limiting the global temperature rise to 1.5 °C. It maps out a pathway for how the global energy sector can reach net-zero emissions by 2050. The NZE has significantly lower primary energy demand, lower fossil fuel demand, and a higher proportion of renewable energy compared to the SDS and the STEPS. It assumes unprecedented growth in renewables to 80% (26568 GW)of global electricity capacity by 2050, of which solar PV capacity and wind energy will account for 43% (14458 GW) and 25% (8265 GW) of renewable energy respectively[2].

1.2 Engineering design parameters of relevant projects

The engineering parameters of wind and solar PV plant projects, such as the site selection, project scale, layout design of Inter-array grids, export transmission line design, and other engineering parameters for individual projects, vary according to the technical type and specific requirements. Even for the same power plant projects, there could be quite different design choices. We analyze and summarize the most typical engineering designs of these projects, and their future development trends.

Typical Project Size

The average size of these renewable energy projects has grown in recent years. For wind projects, their average size has increased 8-fold, reaching about 380 MW, and is expected to grow further to exceed 1000 MW for offshore wind[3,4]. Based on the average size of global offshore wind power projects from 2014 and 2019 to 2021, we assume that their future average growth trend follows a logarithmic growth model and the average size will be 1185 MW in 2050. The average size of offshore wind farms over the years has far exceeded the average size of their onshore counterparts. This trend is expected to continue over the next few decades. We

take the global average project size of onshore wind farms in 2016 as a starting point of 81 MW[5], and assume that by 2050, the average project size of onshore wind farms will grow at the same rate as their offshore counterparts and thus the final estimate in 2050 is 295 MW. For solar PV projects, we take their average project size of 46MW in Europe and emerging markets in 2017 as a starting point[6], and expected size of 200 MW in 2050 as the target value[7], and then linearly interpolate values in the intermediate period.

Distance of export transmission lines

The export cable length between the power plants and the existing electricity grid is another key element. The worldwide trend is characterized by the increase of distance to the main grid for both installed wind and solar power[4,8]. This is because abundant wind and solar energy are often located far away from population centers, resulting in an increasing distance between the sites of newly planned projects and the existing electricity grid. The related data on average distance to the main grid is very limited, and we thus make some assumptions based on trend prediction in literature reports and combined with our own judgments. Regarding the offshore wind projects, we set the average offshore distance of projects in 2016 at 30 km[4], and the expected value of offshore wind projects in 2050 at 200 km[8–10]. Then we estimate the values during this period by linear interpolation. A sensitivity analysis of these assumptions can be found in the section 1.5. For onshore wind projects, we refer to the relevant technical reports and set the distance from the main grid network at 20 km[11,12]. We assume that by 2050 the average distance between wind and solar plants and the population centers will increase by 25%. Similarly, for the average distance between solar PV projects and the nearest grid connection point, we set this to 15 kilometers, and assume a 25% increase by 2050[13,14].

Inter-array Grids

The layout and topology of inter-array grids for wind and utility-scale solar PV power projects are complex. Taking offshore wind farms as an example, to minimize the length of cable between turbines and the total cable cost, and to reduce cable failures, various layout models have been developed to optimize the array cable routing, such as string structure, radial structure and closed-loop structure[15–17]. We have used an empirical formula [18,19]to estimate the array cable length of a typical wind farm[18,19]. We assume that the inter-array cables of both offshore and onshore wind farms follow the same empirical formula, as follows:

$$l_{arr} = 0.00067 \times c^2 + 14.6 \quad \text{(eq. S1)}$$

Where l_{arr} refers to the required length (unit: km) of inter-array cables and c refers to the capacity (unit: MW) of wind farms. Both 0.00067 and 14.6 are constants that have been empirically derived from the layout of existing parks [18]. For the inter-array cable length of utility-scale solar PV, the cable length coefficient in recent reports[20–23], averaging about 1.9km/MW, is adopted to estimate the total demand for inter-array cables (Table S1).

Transformers & Substations

The rated power size and number of transformers and other electrical equipment in a substation (e.g. switchgear, circuit breaker), also varies with the voltage and power output required for different projects. To simplify the electrical system model, here we refer to the general design principles of wind or solar PV power plant circuits[24,25]: there are two main transformers and other substation equipment for each project, one is for daily use and the other is for backup. The rated power of transformers installed in these power plants is roughly proportional to the installed capacity of the power projects. Based on this assumption, the transformer

specifications installed in wind/solar PV power plants of different types and years are estimated, as detailed in the following section 1.4.2.

Table S1. Review on length coefficient of infield cable of solar PV farm

References	km/MW	Remarks
[20]	9.3 (all included)	including all types of cables: power cables, signal and communication cables, etc. So this value is excluded.
[21]	1.5-2.5(main DC cable)	In 2011-12, a typical solar project used around 3-4 km/MW of main DC cables. This value has now been reduced by around 43 % to 1.5-2.5 km/MW.
[22]	2 (main DC cable)	In 2011, a typical solar project used around 3-4 km/MW of main DC cables. This value has now been reduced to 2 km/MW.
**Value of DC cable adopted in the study**	1.5 (main DC cable)	The shorter the cable, the less energy is lost. The related development trend is to optimize the topology of cabling and shorten the length of cables. To reflect future trends, we use the smallest value available.
[23]	0.75 (AC cable)	The value is derived from the paper in 2009.
**Value of AC cable adopted in the study**	0.42 (AC cable)	The latest data available is more than ten years ago. In order to reflect technological changes, we assume that the current AC cable coefficient of solar PV farms has also dropped by 40% compared to before based on the trend of the DC cables over time.
**Total length**	**1.92 (DC + AC cable)**	In general, there are three types of cables used in a PV system: DC string cables, DC main cables, and solar AC cables. DC string cables are pre-assembled into the panels by the PV panel manufacturer, so they usually cannot be changed. While DC main cables and solar AC cables both are larger power collector cables, which are installed in the transmission grid development stage. So we only consider the latter two cables.

1.3 Metal composition and intensities for transmission lines

Typical power cables and transformers used in offshore wind, on-shore wind and utility-scale solar PV projects have their own characteristics. Even the power grid with the same technology has its own characteristics in the selection of infield cables and outfield cables. This section presents the material intensities of electrical grid components for different energy technologies and different parts of the electrical grids.

Table S2. The typical metal intensity of transmission lines of inter-array grids and export lines for wind (offshore & onshore) and solar PV projects (for the details, please see Table S3 - S21).

Categories	Cu (t/km)	Al (t/km)	Steel (t/km)
**Cables**			
inter-array submarine cable (offshore wind) (33 to 66 kV)	8.2	0.7	11.2
export submarine cable (offshore wind; 100 to 320 kV; DC and AC)	15.5	1.2	16.8
inter-array cable (onshore wind; 10 to 66 kV)	2.1	0.4	/
export cable (onshore; 110 to 500 kV)	0.4	6.7	4.9
solar cables (solar PV; DC, up to 1.5 kV; AC, 0.6 to 36 kV)	2.2	0.4	/
export cable (solar PV; assuming the same as the export cables for onshore wind farms)	0.4	6.7	4.9
**Transformers & Substation equipment**			
	Cu (t/100 MVA)	Al (t/100 MVA)	Steel (t/100 MVA)
transformers (offshore wind)	9.7	1.3	164
transformers (onshore wind)	17.3	0.37	47.6
transformers (utility-scale solar	17.3	0.37	47.6

PV)			
Circuit breakers	0.5	2.5	1.4
Switchgears	0.9	6.7	1.7

The electrical elements of power transmission for both wind farms and solar PV plants included in our research consist of power cables (array cables and export cables), transformers, and substations. The electrical design and selection of related electrical elements in power transmission systems vary by individual projects and different renewable technologies. Even for the same power project, the potential design of the transmission system and the selection of its electrical element are also not unique and may change with a series of complex factors including transmission voltages, installation specifications, environmental conditions in project terrain, investment cost, expected operating objectives, etc. It is therefore not possible to provide accurate and conclusive design guides for future transmission projects to meet their specific needs. As a result, the actual metal intensities contained in these transmission facilities also is difficult to determine.

On the other hand, data on the metal intensity of electricity transmission systems used in electricity transmission networks are very limited and rough. Out of business privacy considerations, most cable and auxiliary manufacturers do not disclose the exact material intensity of their products. The available metal intensity data in the related studies are either too general and only represent the situation of main grids, or too detailed and represent only the situation of a specific transmission project, which cannot reflect the characteristics of the expansion of the power transmission system due to the introduction of renewable technology. In fact, the electricity transmission systems of power plants for each renewable energy technology have their common features. To show such features, as well as future development trends of the power transmission system of renewable power technology, we determined the most representative metal intensity of various power transmission systems based on extensive

literature, technical reports, product manuals, and our assumptions and estimates. We elaborated on this in the following section.

In addition, we assume there will be no revolutionary breakthroughs in transmission technology in the future. It is generally believed that with the continuous increase of transmission distance and output power, high-voltage DC and flexible DC transmission will be more common than AC transmission[26]. However, such changes generally have no significant impact on the cable metal composition. Besides, other power transmission technologies such as high-temperature superconductors are still also on the rise, and it is expected to be difficult to widely apply in the short term due to their high costs and technical challenges[27,28]. Accordingly, the impact of transmission technology on the demand for metals thus is not considered in this study.

Furthermore, some studies also have estimated the material intensities of power cables and transformers for the whole electrical grid networks. By comparison, it is found that the material density of cables or transformers used in these studies is higher than that in this study. The difference is understandable. This is because the electrical grids concerned in this study are the inter-array and export transmission lines for wind and photovoltaic renewable energy projects. The smaller grid transmission capacity of individual renewable energy projects tends to correspond to smaller conductor diameters and thus smaller average material intensities. However, current studies do not subdivide the power grids corresponding to different technical categories but focus on all power grids, which may include dedicated transmission lines (e.g. China's West-to-East Power transmission project). This makes the load capacity of the hypothetical circuit larger, and thus the larger corresponding average material intensities.

1.3.1 Metal intensities of transmission cables and lines

Offshore

Offshore wind farm cable elements include array cables that connect each wind turbine and bring power from turbines to a substation platform and export cable that transmits power from substation to landfall. A submarine power cable is a transmission cable for carrying electric power below the surface of the water, which is widely used in offshore wind farms. Compared to overhead and landlines, submarine power cables are usually equipped with single or double armor (e.g. stainless steel wire armor) to protect cables from seawater corrosion and external impact, but this also results in a larger diameter and heavier mass of submarine cables. Currently, due to the diverse application, there is a large number of submarine power cables available on the market, with various conductor choices, shapes, sizes, lengths, etc. The choice of cable used as array cable and export cable is usually very different because of the difference in capacity load, rated voltage, cross-sectional area, cable length, and other aspects, and this also leads to the difference in their metal intensities. To get a general idea about the different characteristics of these two types of power cables, as well as their most generic metal intensities, we discuss the most important properties of these two types of submarine power cables.

Array cables: The infield voltage values of offshore wind parks that have been built and operated generally range from 20 kV to 35 kV[29–32]. Among them, 33 kV (rated at 36 kV) is the most commonly used voltage level in inter-array grids, which is called standard voltage in some reports[32–34]. Meanwhile, since the average capacity of wind farms and the average single capacity of each wind turbine are going to increase, 66 kV infield-array systems with higher voltage level is expected to be applied for future new projects. Operating at this increased voltage could result in lower power losses, fewer array cabling, and potential cost savings that come with it, and this technology is expected to be applied in more new-built offshore wind projects[32,35–38]. In addition, some offshore wind projects use an infield voltage of 45 kV for

their inter-array grids. Therefore, here we assume that the grid voltage in the offshore wind farm will be within 33-66 kV range for the next 30 years.

After the indicative array power systems were established, the most representative corresponding cable conductors and the associated conductor cross-sectional areas were also determined. Regarding the choice of conductor material for submarine cables, generally, copper conductor cable is most commonly used for connecting offshore wind farms[39]. This is because copper conductors offer a more stable structure in the seabed and longer life cycles in harsh operating conditions. At the same time, due to lower raw material costs and installation costs, some utilities see the economic opportunities of using aluminum as submarine cable conductors and have begun exploring the potential of aluminum submarine cables[40,41]. However, It is still not clear whether aluminum submarine cables will be used widely for future offshore wind projects, and some reports suggest that copper submarine cables are likely to remain dominant in the future[42]. Here, we used the 2016 penetration rate of aluminum in power cables (approximately 16%) as the proportion of aluminum submarine cables used in offshore wind power projects in the following decades[43], and the other 84% of submarine cables are copper conductors. In addition to conductors, the metal parts of the submarine power cable may also be sheath and armor, which are added to prevent water ingression and protect the cable from mechanical loading as well as taking care of the tension stability respectively[44,45]. The armor consists of metal sire, commonly galvanized steel; the sheath material can be lead, copper, copper, and sometimes polymer. Because of the limited data, we assumed all submarine power cables in this study are made of the lead sheath and steel armor.

In terms of conductor size, two other main factors affecting the metal intensity of the cable need to be considered. One is the number of conductor cores per cable, and the other is the cross-sectional area of the conductor, which are also indispensable parameters for cable specification. Currently, the majority of offshore wind power projects use three-core AC (alternating current)

cables in the infield circuit and rarely use single-core cables. There is no other related technological innovation to date, it is expected that three-core cables will still occupy the main market of array submarine power cables in the future. The array submarine power cable usually has multiple cross-sectional areas along the route of array electrical systems[46]. This is because array submarine cables in different parts of the infield electrical system carry different amounts of electricity. Generally the closer the array cable is to the substation, the more power it needs to carry and the greater the cross-sectional area of the conductors. Our investigation of related OWFs reports and literature indicates that the cross-sectional areas of array submarine power cables are usually in a wide range vary from 95mm^2 to over 1000mm[245,47–49]. Based on the above discussion, the characteristics of typical submarine power cables used in the array power system of OWFs have been identified. With reference to these cable characteristics and based on relevant literature and technical product reports, the metal densities of typical array power cables are estimated.

Taking these factors into consideration, we collect the data on submarine cables from publicly disclosed information from cable manufacturers or offshore wind project operators, and relevant literature reports. Then we take their average value as the metal intensity of infield cables of offshore wind farms in the next thirty years, as shown below.

Table S3. Metal intensities of Cu submarine power cables for inter-array grids. Note it is common to supply a standard voltage-rated cable for a specific application. For this study, we usually refer to 18 / 30 (36) as the common term '33 kV' as nominal system voltage, similarly refer to 36 / 60-69 (72.5) as the common term '66 kV' and so on.

Sources	Voltage Level (kV)	Cross-sectional area (mm^2)	Total weight in the air (t/km)	Copper		Aluminum		Steel		Lead	
				t/km	%wt	t/km	%wt	t/km	%wt	t/km	%wt
[50]	33 kV	3*95	17.2		11%				50%		28%
		3*150	19.9		15%				47%		26%
		3*240	24.7		21%				43%		26%
		3*400	30.1		27%				33%		31%
		3*630	40.0		39%				32%		20%
[51]	/	/			22%				46%		19%
[52]	18/33 kV	3*95	30.2								
		3*120	32.4								
		3*150	32.8								
		3*185	37.2								
		3*240	40.9								
		3*300	45.2								
		3*400	51.1								
		3*500	56.9								
[53]	33 kV	3*95	19.5								
		3*120	20.7								
		3*150	22.1								
		3*185	23.6								
		3*240	25.9								
		3*300	28.3								
		3*400	32.0								
		3*500	36.0								
		3*630	40.9								
		3*800	47.2								
	45 kV	3*95	20.8								
		3*120	22.3								
		3*150	24.4								
		3*185	26.2								
		3*240	29.5								
		3*300	32.9								

Sources	Voltage Level (kV)	Cross-sectional area (mm^2)	Total weight in the air (t/km)	Copper		Aluminum		Steel		Lead	
				t/km	%wt	t/km	%wt	t/km	%wt	t/km	%wt
		3*400	37.9								
		3*500	43.2								
		3*630	49.7								
		3*800	58.6								
		3*1000	68.1								
	66 kV	3*95	21.6								
		3*120	23.8								
		3*150	25.7								
		3*185	28								
		3*240	31.3								
		3*300	34.3								
		3*400	39.2								
		3*500	45.4								
		3*630	52								
		3*800	60.1								
		3*1000	70.7								
[54]	33 kV	3*95	13.6		22%				41%		
		3*120	14.8		25%				39%		
		3*150	15.9		28%				37%		
		3*185	17.6		31%				36%		
		3*240	20.0		35%				33%		
		3*300	22.4		38%				31%		
		3*400	26.3		42%				29%		
		3*500	31.1		45%				27%		
		3*630	37.1		48%				26%		
[32]	66 kV	3*630	40								
[55]	33 kV	3*95	16.5								
		3*120	17.8								
		3*150	19.3								
		3*185	20.8								

Sources	Voltage Level (kV)	Cross-sectional area (mm^2)	Total weight in the air (t/km)	Copper		Aluminum		Steel		Lead	
				t/km	%wt	t/km	%wt	t/km	%wt	t/km	%wt
		3*240	23.2								
		3*300	25.6								
		3*400	29.9								
		3*500	34.4								
		3*630	39.8								
		3*800	47.3								
	36/66 (72.5)	3*150	36.5								
		3*800	72.3								
[56]	26/35 kV	3*95	23								
		3*120	24.6								
		3*150	26.2								
		3*185	28.2								
		3*240	31.1								
		3*300	34.2								
		3*400	39.3								
		3*300, 3*800	47								
[57]	33~66 kV	3*120	20								
		3*800	**51**								
**Average**	**/**	**/**	**32.8**	**9.8**	**30%**	**/**	**/**	**12.0**	**37%**	**8.2**	**25 %**

Table S4. Metal intensities of Al submarine power cables for inter-array grids. Due to the limited data available, we assume that the material contents (wt%) of multiple metals in aluminum conductor cables are the same as those reported in [58](Al: 13.9 wt%; Steel: 23 wt%; Pb: 33.9 wt%) for 3- core aluminum-conductor submarine power cables. On this basis, combining with the total weight of the cable, the material content of the cable is converted to material density (t/km).

Sources	Voltage Level (kV)	Cross-sectional area (mm^2)	Total weight in the air (t/km)	Aluminum		Steel		Lead	
				t/km	%wt	t/km	%wt	t/km	%wt
[34]	66 kV	3*800	57.9						
		3*800	39.4						
		3*800	36.2						
[53]	33 kV	3*95	17.7						
		3*120	18.4						
		3*150	19.3						
		3*185	20.1						
		3*240	21.4						
		3*300	22.6						
		3*400	24.6						
		3*500	26.7						
		3*630	29.2						
		3*800	32.2						
	45 kV	3*95	19.1						
		3*120	20.0						
		3*150	21.6						
		3*185	22.7						
		3*240	25.0						
		3*300	27.3						
		3*400	30.4						
		3*500	33.8						
		3*630	37.8						

Sources	Voltage Level (kV)	Cross-sectional area (mm^2)	Total weight in the air (t/km)	Aluminum		Steel		Lead	
				t/km	%wt	t/km	%wt	t/km	%wt
		3*800	43.5						
		3*1000	49.3						
	66 kV	3*95	19.8						
		3*120	21.6						
		3*150	22.9						
		3*185	24.5						
		3*240	26.8						
		3*300	28.7						
		3*400	31.7						
		3*500	36.0						
		3*630	40.1						
		3*800	45.1						
		3*1000	51.8						
**Average**		/	**29.9**	**4.2**	**13.9* wt%**	**6.9**	**23.0* wt%**	**10.1**	**33.9* wt%**

Table S5. Metal intensities of typical array submarine cables for OWFs. As discussed above, we assume that copper conductors will account for 84% of the submarine power cable market and aluminum conductor cable for 16% in the future. Then we use the weighted average of the material densities of these two types of array cables to represent the amount of material required for each kilometer of array submarine power cables in the coming decades.

	Penetration rate (%)	Cu (t/km)	Al (t/km)	Steel (t/km)	Pb (t/km)	Total weight (t/km)
Cu array submarine power cable	84	9.8	/	12.4	8.4	33.4
Al array submarine power cable	16	/	4.2	8.1	9.6	29.8
The Weighted average of array submarine power cable	**100%**	**8.2**	**0.7**	**11.2**	**8.5**	**32.3**

Export cable: export submarine power cables that transmit electricity from the offshore substation to land are of similar design with the array submarine power cables but for higher voltage to carry more energies. This also causes a much greater conductor diameter of the export cable than that of the array cable. The voltage level of export submarine power cable for offshore HVAC (High Voltage Alternating Current) transmission is typically between 100 and 320 kV[42,45,59]. When the distance to shore gets longer, HVDC (High Voltage Direct Current) transmission technology and associated submarine power cables are preferred due to lower electrical losses and lower costs[60]. The voltage level of HVDC cables for export cabling of OWFs can be up to 525 kV[59]. In terms of conductor material selection, we assume that the penetration rate of conductor materials of the export cable is the same as that of array cables: copper conductor submarine cables and aluminum conductor cables account for 84% and 16% of the submarine power cable market respectively. Regarding conductor size, the AC (Alternating Current) cable commonly has 3-core conductors, whereas the DC (Director

Current) cable is 1-core conductors[45]. Due to the lack of relevant data, here we assume that the possibility of using AC and DC technologies in the export electrical system of OWFs in the future is equal, which means that the probability of laying a 1-kilometer export submarine power cab with AC and DC cables is 50% respectively. The cross-sectional areas of submarine power cables for export cabling of OWFs commonly range from 400 to 1200 mm2[61], and in some cases, it can be up to 1400 mm[257]. In combination with the above discussion, the metal densities of typical export submarine power cables are estimated as below.

Table S6. Metal intensities of Cu submarine power cables for export electrical systems.

**AC cables**									
Sources	Voltage Level (kV)	Cross-sectional area (mm^2)	Total weight in the air (t/km)	Copper		Aluminum		Steel	
				t/km	Wt%	t/km	Wt%	t/km	Wt%
[50]	132 kV	3*800	74.0		27.0%				35%
[51]	132 kV	/	67		28%				27%
[58]	/	3*1000	101		27.9%				20.5 %
[52]	127/200 kV	3*400	113.2						
		3*500	121.9						
		3*630	127.2						
		3*800	135.0						
		3*1000	143.5						
		3*1200	156.7						
[62]	132 kV	3*800	78						
		3*1000	102						
		3*1200	110						
	275 kV	3*800	101						
		3*1000	112						
		3*1200	125						
[53]	110 kV	3*400	47.2						

Sources	Voltage Level (kV)	Cross-sectional area (mm^2)	Total weight in the air (t/km)	Copper		Aluminum		Steel	
				t/km	Wt%	t/km	Wt%	t/km	Wt%
		3*500	53.0						
		3*630	60.7						
		3*800	69.5						
		3*1000	80.5						
	132 kV	3*400	51.1						
		3*500	58						
		3*630	65.2						
		3*800	74						
		3*1000	85.4						
	150 kV	3*400	60.5						
		3*500	65.1						
		3*630	69.7						
		3*800	79.8						
		3*1000	90.5						
	240 kV	3*500	81.3						
		3*630	86.7						
		3*800	95.3						
		3*1000	104						
	275 kV	3*500	84.7						
		3*630	88.9						
		3*800	97.6						
		3*1000	106.3						
[57]	220 ~290 kV (more commonly)	3*1400	125						
**Average**	/	/	**90.1**	**25.1**				**25.0**	
**DC cables**									

Sources	Voltage Level (kV)	Cross-sectional area (mm^2)	Total weight in the air (t/km)	Copper		Aluminum		Steel	
				t/km	Wt%	t/km	Wt%	t/km	Wt%
		500	29.4						
		630	30.1						
		800	30.1						
		1000	32.4						
		1200	35.8						
	250 KV	400	28.4						
		500	31.8						
		630	34.4						
		800	36.8						
		1000	40.4						
		1200	44.3						
	320 kV	800	50.6						
		1000	54.6						
		1200	56.5						
[57]	/	/	60.0						
[58]	300 kV	1300	37.0		31.4%				26.2 %
[63]	240 kV	630	26.0						
**Average**	**/**	**/**	**38**	**11.9**				**10.0**	
**Weighted average**			**64.5**	**18.5**				**17.5**	

Table S7. Metal intensities of Al submarine power cables for export electrical systems.

AC cables							
Sources	Voltage Level (kV)	Cross-sectional area (mm^2)	Total weight in the air (t/km)	Aluminum		Steel	
				t/km	Wt%	t/km	Wt%
[53]	110 kV	3*400	39.7				
		3*500	43.6				
		3*630	48.8				
		3*800	54.4				
		3*1000	61.6				
	132 kV	3*400	43.6				
		3*500	48.6				
		3*630	53.3				
		3*800	59.0				
		3*1000	66.6				
	150 kV	3*400	52.9				
		3*500	55.7				
		3*630	57.8				
		3*800	64.7				
		3*1000	71.6				
	240 kV	3*500	71.8				
		3*630	74.9				
		3*800	80.2				
		3*1000	85.1				
	275 kV	3*500	75.3				
		3*630	77.0				
		3*800	82.5				
		3*1000	87.4				
[62]	132 kV	3*800	61				
		3*1000	68				
		3*1200	74				
	275 kV	3*800	84				
		3*1000	90				
		3*1200	97				

Sources	Voltage Level (kV)	Cross-sectional area (mm^2)	Total weight in the air (t/km)	Aluminum		Steel	
				t/km	Wt%	t/km	Wt%
**Average**	**/**	**/**	**66.5**	**9.3**		**15.3**	
DC cables							
[58]	300 kV	2000*	**34**	**5.6**	**16.5 %**	**10.6**	**31.2%**
**Weighted average**			**50.3**	**7.4**		**13.0**	

Table S8. Metal intensities of typical export submarine cables for OWFs. Here we estimate the material intensity of a typical export submarine cable using a similar method to that of a typical array submarine power cable. See Table s3 for a detailed description.

	Penetration rate (%)	Cu (t/km)	Al (t/km)	Steel (t/km)	Total weight (t/km)
Cu export submarine power cable	84	18.5	/	17.5	64.5
Al export submarine power cable	16	/	7.4	13.0	50.3
The Weighted average of the export submarine power cable	**100%**	**15.5**	**1.2**	**16.8**	**62.2**

Onshore

Similar to the power transmission system of offshore wind farms, power transmission systems of onshore wind farms mainly consist of infield-array grid systems and export grid systems. The inter-array grid system connects each wind turbine and collects electricity from wind turbines to the substation, and export grid systems transport power from the onshore wind farms to the main transmission network or directly to the nearest distribution network. Generally, the cable used for the infield-array grid system of onshore wind farms is underground cable, while the cable for export grid system can be either

underground cable or overhead cables. The underground cable is buried in the ground and equipped with lead sheet and armoring, which protects against moisture and mechanical injury. While overhead transmission cable uses bare conductors and these conductors are placed at a height from the ground. The choice of cable for inter-array grid systems and export grid systems of onshore wind farms also varies with different projects, depending on expected nominal voltage, power load, investment cost, etc., but they do also have their characteristics. We estimated the metal intensities of these cables based on their features as follows.

Array cable: We adopt similar analysis steps as for offshore wind farm cables to determine the generic characteristic parameters of different parts of the cable system, then estimate the typical cable intensities for array cable as well as export cable for onshore wind farms based on these characteristics.

The voltage level of the array cable system of an onshore wind farm is commonly in the range of 10 to 66 kV[64]. The conductor material of these array cables could be copper or aluminum.

Table S9. Metal intensities of Cu underground power cables for array electrical systems of onshore wind farms. Note: here we used the mass content of each material in the underground copper cable for the export system[51] to estimate the metal intensities of the array cable.

Sources	Voltage Level (kV)	Cross-sectional area (mm^2)	Total weight in the air (t/km)	Copper		Aluminum		Steel	
				t/km	%wt	t/km	%wt	t/km	%wt
[51]	22 kV		5.1						
[65]	12 kV	95*3	8.0						
		120*3	9.1						
		150*3	10.3						
		185*3	11.7						
		240*3	15.0						
		300*3	17.5						
		400*3	20.9						
	12 kV	95	2.0						
		120	2.3						
		150	2.7						
		185	3.1						
		240	3.8						
		300	4.5						
		400	5.4						
		500	6.7						
		630	8.2						
		800	12.2						
		1000	12.5						
	22 kV	95	2.4						
		120	2.7						
		150	3.1						
		185	3.5						
		240	4.2						
		300	5.1						
		400	6.0						

Sources	Voltage Level (kV)	Cross-sectional area (mm^2)	Total weight in the air (t/km)	Copper		Aluminum		Steel	
				t/km	%wt	t/km	%wt	t/km	%wt
		500	7.2						
		630	8.8						
		800	10.8						
		1000	13.1						
		95*3	9.5						
		120*3	11.5						
		150*3	12.8						
		185*3	14.4						
		240*3	16.8						
		300*3	19.5						
		400*3	23.0						
	36 kV	95	2.8						
		120	3.2						
		150	3.7						
		185	4.2						
		240	4.8						
		300	5.6						
		400	6.6						
		500	7.8						
		630	9.4						
		800	11.6						
		1000	13.4						
	36 kV	95*3	7.6						
		120*3	8.6						
		150*3	9.6						
		185*3	11.0						
		240*3	13.2						
		300*3	15.6						
		400*3	18.7						
[66]	66 kV	185	13						
		240	14						

Sources	Voltage Level (kV)	Cross-sectional area (mm^2)	Total weight in the air (t/km)	Copper		Aluminum		Steel	
				t/km	%wt	t/km	%wt	t/km	%wt
		300	14						
		400	15						
		630	16						
		600	18						
		800	20						
		1000	22						
		1200	24						
**Average**			**10.1**	**2.4**	**24%**				

Table S10. Metal intensities of Al underground power cables for array electrical system of onshore wind farms

Sources	Voltage Level (kV)	Cross-sectional area (mm^2)	Total weight in the air (t/km)	Copper		Aluminum		Steel	
				t/km	%wt	t/km	%wt	t/km	%wt
	50 kV	/	3.3	0.5	15.2%	1.0	30.3%		
[67]	33 kV		3.2		9%		42%		
[65]	12 kV	95	1.4						
		120	1.5						
		150	1.8						
		185	2.0						
		240	2.3						
		300	2.6						
		400	3.0						
		500	3.6						
		630	4.2						
		800	5.1						
		1000	6.0						
	12 kV	95*3	6.2						
		120*3	6.8						
		150*3	7.4						
		185*3	8.3						
		240*3	10.4						
		300*3	11.7						
		400*3	13.4						
	36 kV	95	1.7						
		120	1.9						
		150	2.0						
		185	2.2						
		240	2.5						
		300	2.8						
		400	3.2						
		500	3.6						
		630	4.2						

Sources	Voltage Level (kV)	Cross-sectional area (mm^2)	Total weight in the air (t/km)	Copper		Aluminum		Steel	
				**t/km**	**%wt**	**t/km**	**%wt**	**t/km**	**%wt**
		800	5.1						
		1000	6.0						
[66]	66 kV	185	12						
		240	12						
		300	12						
		400	13						
		630	13						
		600	14						
		800	15						
		1000	15						
		1200	16						
**Average**			**6.5**	**0.80**	**12%**	**2.34**	**36.2 %**		

Table S11. Metal intensities of typical array underground cables for onshore wind farms. As discussed above, we assume that copper conductors will account for 84% of the submarine power cable market and aluminum conductor cable for 16% in the future. Then we use the weighted average of the material densities of these two types of array cables to represent the amount of material required for each kilometer of array power cables in the coming decades.

	Penetration rate (%)	Cu (t/km)	Al (t/km)	Steel (t/km)	Pb (t/km)	Total weight (t/km)
Cu array underground power cable	84	2.4			3.9	10.1
Al array underground power cable	16	0.78	2.34			6.5
The Weight average of array underground power cable	**100%**	**2.1**	**0.4**		**3.4**	**9.5**

Table S12. Metal intensities of Cu underground power cables for export electrical systems of onshore wind farms.

Sources	Voltage Level (kV)	Cross-sectional area (mm²)	Total weight in the air (t/km)	Copper		Aluminum		Steel	
				t/km	%wt	t/km	%wt	t/km	%wt
[68]	150 kV	/	36.1	8.6	23.8%				
[69]	132 kV		36.1		24%				0.0
[66]	110 kV	240	15						
		300	16						
		400	17						
		630	18						
		800	23						
		1000	23						
		1200	24						
	132 kV	300	16						
		400	16						
		500	17						
		630	19						
		800	21						
		1000	23						
		1200	25						
		1600	30						
	225 kV	400	23						
		500	24						
		630	26						
		800	27						
		1000	30						
		1200	32						
		1600	37						
	275 kV	500	25						
		630	26						

Sources	Voltage Level (kV)	Cross-sectional area (mm^2)	Total weight in the air (t/km)	Copper		Aluminum		Steel	
				t/km	%wt	t/km	%wt	t/km	%wt
		800	28						
		1000	30						
		1200	32						
		1600	38						
	345 kV	500	25						
		630	26						
		800	28						
		1000	31						
		1200	32						
		1600	38						
	400 kV	500	34						
		630	35						
		800	36						
		1000	38						
		1200	40						
		1600	46						
	500 kV	1000	42						
		1200	42						
		1600	43						
		2000	48						
**Average**			**29.1**	**6.9**	**24%**				

Table S13. Metal intensities of Al underground power cables for export electrical systems of onshore wind farms.

Sources	Voltage Level (kV)	Cross-sectional area (mm^2)	Total weight in the air (t/km)	Copper		Aluminum		Steel	
				t/km	%wt	t/km	%wt	t/km	%wt
[50]	132 kV	3*800			0.9%		35.2%		
[67]	110 kV		7.7		4.0%		34%		
[66]	110 kV	240	13						
		300	13						
		400	14						
		630	14						
		800	15						
		1000	16						
		1200	16						
	132 kV	300	14						
		400	14						
		500	14						
		630	15						
		800	16						
		1000	15						
		1200	17						
		1600	19						
	225 kV	400	21						
		500	21						
		630	22						
		800	23						
		1000	24						
		1200	24						
		1600	26						
	275 kV	500	22						
		630	22						
		800	23						
		1000	23						

**Sources**	**Voltage Level (kV)**	**Cross-sectional area (mm^2)**	**Total weight in the air (t/km)**	**Copper**		**Aluminum**		**Steel**	
				**t/km**	**%wt**	**t/km**	**%wt**	**t/km**	**%wt**
		1200	24						
		1600	27						
	345 kV	500	22						
		630	23						
		800	23						
		1000	24						
		1200	25						
		1600	27						
	400 kV	500	31						
		630	31						
		800	31						
		1000	32						
		1200	32						
		1600	35						
	500 kV	1000	36						
		1200	36						
		1600	37						
		2000	38						
**Average**			**22.6**	**0.6**	**2.5%**	**7.8**	**34.6 %**		

Table S14. Metal intensities of typical export underground cables for onshore wind farms.

	Penetration rate (%)	Cu (t/km)	Al (t/km)	Steel (t/km)	Total weight (t/km)
Cu array underground power cable	84	6.9			29.1
Al array underground power cable	16	0.6	7.8		22.6
The Weight average of array underground power cable	**100%**	**6.0**	**1.2**		**28.1**

Table S15. Metal intensities of typical overhead power cables for export electrical systems of onshore wind farms.

Sources	Voltage Level (kV)	Cross-sectional area (mm^2)	Total weight in air (t/km)	Copper		Aluminum		Steel	
				t/km	%wt	t/km	%wt	t/km	%wt
[50]	132 kV	/	/			7.3		12.9	
[68]	150 kV					1.1		3.3	
	400 kV					11.9		4.3	
[70]	220 kV					6.6		2.5	
	300 kV					8.4		3.4	
**Average**						**7.1**		**5.3**	

Table S16. Metal intensities of power cables for export electrical systems of onshore wind farms. The share of overhead lines and underground cables in connections to the main grid must also be considered. Here we adopted the ratio of overhead cables to underground cables in the European power network in 2016 (13:1)[71] to indicate that for laying one kilometer of export cables, the probability of using overhead cables is 93%, while underground cables are 7%.

	Portion(% km)	Cu (t/km)	Al (t/km)	Steel (t/km)
Underground power cable for export cabling	7%	**6.0**	**1.2**	
Overhead power cable for export cabling	93%		**7.1**	**5.3**
Estimation of power cable for export cabling	**100**	**0.4**	**6.7**	**4.9**

Utility-scale Solar PV

Similar to wind power farms, the cable transmission system of utility solar PV plants includes in-field array cable for the connection between the PV components, and a dedicated export

cable line (usually called a generation tie or “gen-tie”) for transferring output electricity to the existing substation or main grid. According to the characteristics of utility solar PV plants, we discussed its typical cable intensity.

Array cable: The utility solar PV plants use both DC (direct current) and AC (alternate current) power to MV/ HV transformers and substations. Cables used are typically three types. DC cables are required to interconnect solar modules, string arrays, and array combiner boxes up to the inverter in the DC circuit, usually with low voltage levels (smaller than 1.5 kV). While two types of AC power cables are employed to transfer the AC output of inverters to LV (low voltage)/MV (medium voltage: (10 ~ 45 kV)) transformers[72,73]**.** Similarly, copper and aluminum are the two most common conductor materials used in solar cables. The conductor metal of solar DC cable is bare copper or tinned copper, rarely aluminum. While the conductor of the AC cable can be copper or aluminum.

Same as other power cables we discussed before, we again apply the same assumption of the market penetration rate of copper and aluminum conductors (84% and 16% respectively). Based on these characteristics of solar cables, the metal intensity of typical solar PV cables are estimated as follows.

Table S17. Metal intensities of solar DC cables for array electrical systems.

Sources	Voltage Level (kV)	Cross-sectional area (mm^2)	Total weight in air (t/km)	Cu (t/km)
[74]	0.6/1 kV	1*2.5	0.042	0.024
	0.6/1 kV	1*4	0.06	0.038
	0.6/1 kV	1*6	0.082	0.058
	0.6/1 kV	1*10	0.123	0.096
	0.6/1 kV	1*16	0.19	0.154
	0.6/1 kV	1*25	0.285	0.24
	0.6/1 kV	1*35	0.376	0.336
	0.6/1 kV	1*50	0.53	0.48
	0.6/1 kV	1*70	0.745	0.672
	0.6/1 kV	1*95	0.96	0.912
	0.6/1 kV	1*120	1.22	1.15
	0.6/1 kV	1*150	1.55	1.44
	0.6/1 kV	1*185	1.93	1.78
	0.6/1 kV	1*240	2.55	2.30
[75]	0.6 kV	1*600 kcmil	3.08	2.80
	0.6 kV	1*750 kcmil	3.76	3.45
	0.6 kV	1*1000 kcmil	4.95	4.60
**Average**			**1.3**	**1.2**

Table S18. Metal intensities of solar AC cables (low voltage) for array electrical systems.

Cu conductor				
Sources	Voltage Level (kV)	Cross-sectional area (mm^2)	Total weight in air (t/km)	Cu (t/km)
[74]	0.6/1 kV	1*25	0.36	0.24
	0.6/1 kV	1*35	0.47	0.34
	0.6/1 kV	1*50	0.62	0.48
	0.6/1 kV	1*70	0.81	0.67
	0.6/1 kV	1*95	1.11	0.91
	0.6/1 kV	1*120	1.36	1.15
	0.6/1 kV	1*150	1.67	1.44
	0.6/1 kV	1*185	2.05	1.78
	0.6/1 kV	1*240	2.63	2.30
	0.6/1 kV	1*300	3.2	2.88
	0.6/1 kV	1*400	4.15	3.84
	0.6/1 kV	1*500	5.2	4.8
	0.6/1 kV	1*630	6.65	6.05
	0.6/1 kV	3* 25	1.32	0.72
	0.6/1 kV	3* 35	1.45	1.01
	0.6/1 kV	3* 50	1.85	1.44
	0.6/1 kV	3* 70	2.45	2.02
	0.6/1 kV	3* 95	3.3	2.74
	0.6/1 kV	3* 120	4.1	3.46
	0.6/1 kV	3* 150	4.9	4.32
	0.6/1 kV	3* 185	6.5	5.33
	0.6/1 kV	3* 240	8.3	6.91
	0.6/1 kV	4* 25	1.64	0.96
	0.6/1 kV	4* 35	1.76	1.34
	0.6/1 kV	4* 50	2.35	1.92
	0.6/1 kV	4* 70	3.1	2.69
	0.6/1 kV	4* 95	4.25	3.65
	0.6/1 kV	4* 120	5.3	4.61
	0.6/1 kV	4* 150	6.4	5.76
	0.6/1 kV	4* 185	8.5	7.11

	0.6/1 kV	4* 240	11	9.22
**Average**			**3.5**	**3.0**

Al conductor				
Sources	Voltage Level (kV)	Cross-sectional area (mm^2)	Total weight in air (t/km)	Al (t/km)
[74]	0.6/1 kV	1*35	0.24	0.1
	0.6/1 kV	1*50	0.36	0.15
	0.6/1 kV	1*70	0.41	0.20
	0.6/1 kV	1*95	0.57	0.28
	0.6/1 kV	1*120	0.69	0.35
	0.6/1 kV	1*150	0.81	0.44
	0.6/1 kV	1*185	0.979	0.54
	0.6/1 kV	1*240	1.25	0.70
	0.6/1 kV	1*300	1.40	0.87
	0.6/1 kV	1*400	1.90	1.12
	0.6/1 kV	1*500	2.60	1.45
	0.6/1 kV	1*630	2.78	1.83
	0.6/1 kV	4* 25	0.95	0.30
	0.6/1 kV	4* 35	1.12	0.41
	0.6/1 kV	4* 50	1.15	0.58
	0.6/1 kV	4* 70	1.55	0.82
	0.6/1 kV	4* 95	2.03	1.10
	0.6/1 kV	4* 120	2.40	1.39
	0.6/1 kV	4* 150	3.03	1.74
	0.6/1 kV	4* 185	3.65	2.15
	0.6/1 kV	4* 240	4.8	2.78
	0.6/1 kV	4* 300	5.6	3.5
**Average**			**1.8**	**1.0**

Table S19. Metal intensities of solar AC cables (medium voltage) for array electrical systems.

Cu conductor					
Sources	Voltage Level (kV)	Cross-sectional area (mm^2)	Total weight in the air (t/km)	Cu (t/km)	Al (t/km)
[74]	6/10 kV	1*95	1.60	1.1	/
	6/10 kV	1*120	1.86	1.33	/
	6/10 kV	1*150	2.24	1.62	/
	6/10 kV	1*185	2.58	2.06	/
	6/10 kV	1*240	3.13	2.59	/
	6/10 kV	1*300	3.78	3.16	/
	6/10 kV	1*400	4.67	4.23	/
	6/10 kV	1*500	5.75	5.20	/
	6/10 kV	3*95	5.7	3.00	/
	6/10 kV	3*120	6.7	3.72	/
	6/10 kV	3*150	7.9	4.64	/
	6/10 kV	3*185	9.2	5.65	/
	6/10 kV	3*240	11.45	7.27	/
	6/10 kV	3*300	14.45	9.16	/
	12/20 kV	1*95	1.78	1.1	/
	12/20 kV	1*120	2.07	1.33	/
	12/20 kV	1*150	2.42	1.62	/
	12/20 kV	1*185	2.81	2.06	/
	12/20 kV	1*240	3.36	2.59	/
	12/20 kV	1*300	4.02	3.16	/
	12/20 kV	1*400	4.93	4.23	/
	12/20 kV	1*500	6.05	5.20	/
**Average**			**4.9**	**3.5**	
Al conductor					
	6/10 kV, 12/20 kV, 18/36 kV	1*95	/	0.18	2.76
	6/10 kV, 12/20 kV, 18/36 kV	1*120	/	0.18	3.48
	6/10 kV, 12/20 kV, 18/36 kV	1*150	/	0.28	4.35
	6/10 kV, 12/20 kV, 18/36 kV	1*185	/	0.28	5.37

Sources	Voltage Level (kV)	Cross-sectional area (mm^2)	Total weight in the air (t/km)	Cu (t/km)	Al (t/km)
	6/10 kV, 12/20 kV, 18/36 kV	1*240	/	0.28	6.96
	6/10 kV, 12/20 kV, 18/36 kV	1*300	/	0.28	0.87
	6/10 kV, 12/20 kV, 18/36 kV	1*400	/	0.39	1.16
	6/10 kV, 12/20 kV, 18/36 kV	1*500	/	0.39	1.45
**Average**				**0.3**	**3.3**
**The Weighted average of medium voltage solar AC cable (Cu, 84%; Al, 16%)**				**3.0**	**0.5**

Table S20. Metal intensities of in-field array cable for typical solar PV plants

	Cu (t/km)	Al (t/km)
Solar DC cables	1.2	/
Solar AC cables (Low voltage)	2.5	0.2
Solar AC cables (high voltage)	3.0	0.5
**Average**	**2.2**	**0.4**

Export cable: Unless the solar PV plant is right next to a substation or a transmission network, a dedicated export transmission line has to be constructed. While utility-scale solar PV projects are generally located in less populated, open areas such as suburbs and deserts, where power transmission infrastructure is often weak. The construction of these power plants is thus usually accompanied by the construction of medium and high voltage export transmission lines linked to the main grid. These export cables can be implemented using underground cables or overhead cables depending on specific projects, which is the same as that of onshore wind farms. Here we use the metal intensity of the export cable of onshore wind farms to represent the metal intensity of the export cable of solar PV plants (see Table 14).

1.3.2 Transformers and substation intensity

Power grids usually operate at much higher voltages in the order of tens or hundreds of thousands of volts, transformers are incorporated in renewable energy projects to deliver the required output to the grid. Different sizes of power plants require different specifications of transformers. Generally speaking, the larger the installed capacity of a power plant, the larger the rated load (MVA) of the transformer required, as well as its size and mass. According to the previous (see section 2.3) estimation of future sizes of the power plant, we assume an anticipated offshore wind farm with 1185 MW use transformers rated at 1200 MVA; anticipated onshore wind farm with 294MW use transformers rated at 300 MVA; anticipated utility solar PV plant with 200 MW use transformers rated at 200 MVA.

Table S21. literature of metal intensity for transformers and substations (per 100 MVA)

Onshore wind & Utility solar PV				
Sources	Rated Capacity (MVA)	Cu (t/unit)	Al (t/unit)	Steel(t/unit)
[76]	100 MVA	8		30.6
[77]	20 MVA	8.7	0.09	20.5
	50 MVA	9.03	0.09	35.9
	63 MVA	18.4		35.7
	250 MVA	24.2	2.0	112.7
	500 MVA	40		153.3
[51]	150 MVA	14.5	1.2	65.8
[78]	100 MVA	12.5		
**Average (t/100 MVA)**	/	**17.3**	**0.37**	**47.6**
Offshore wind				
[51]	250 MVA	24.2	3.3	410
**Average (t/100 MVA)**		**9.7**	**1.3**	**164**

Table S22. Estimation of the metal intensity of the transformers for 3 types of typical renewable power plants

Renewable power plants	Rated Capacity (MVA)	Cu (t/unit)	Al (t/unit)	Steel(t/unit)
Offshore wind	1200	116.2	15.8	1968
Onshore wind	300	52	1.1	142.9
Utility solar PV	200	34.7	0.7	95.3

In addition to cables and transformers, the power transmission system also needs other auxiliary power equipment, such as switches, circuit breakers, etc. The equipment is often placed in substations for unified management. When planning renewable power projects, the construction of substations is also considered. The substation further boosts the power collected from renewable power plants for subsequent transmission to the main grid. Substations are also very diverse in size and complexity, and it is impossible to examine the very detailed layout information for different renewable power projects. Here we summarize and estimate the typical metal intensity of some important auxiliary power equipment used in substations, as shown in Table S20. In addition, due to the limited data, we assume that the substations supporting all kinds of renewable energy power plants are the same.

Table S23. The metal intensity of substation equipment

Circuit breaker				
Sources	Variant	Cu (t/unit)	Al (t/unit)	Steel(t/unit)
[76]	Circuit breaker, AIS, 132 kV	0.1	0.2	0.5
	Circuit breaker, AIS, 275/400 kV	0.1	0.2	0.6
	Circuit breaker, GIS, 132 kV	0.9	4.0	1.7
	G Circuit breaker, IS, 275/400 kV	1.8	8.4	3.5

Sources	Variant	Cu (t/unit)	Al (t/unit)	Steel(t/unit)
[77]	Circuit Breaker, Type HECS	0.2	1.8	1.4
	Live Tank Circuit Breaker	0.1	0.2	0.5
**Average**		0.5	2.5	1.4
Switchgear				
[77]	300 kV, GIS	0.9	4.1	1.3
	420 kV, GIS	0.9	9.2	2.1
**Average**		0.9	6.7	1.7

GIS: Gas Insulated Switchgear； AIS： Air Insulated Switchgear

1.4 Survival curves of the electrical grid systems

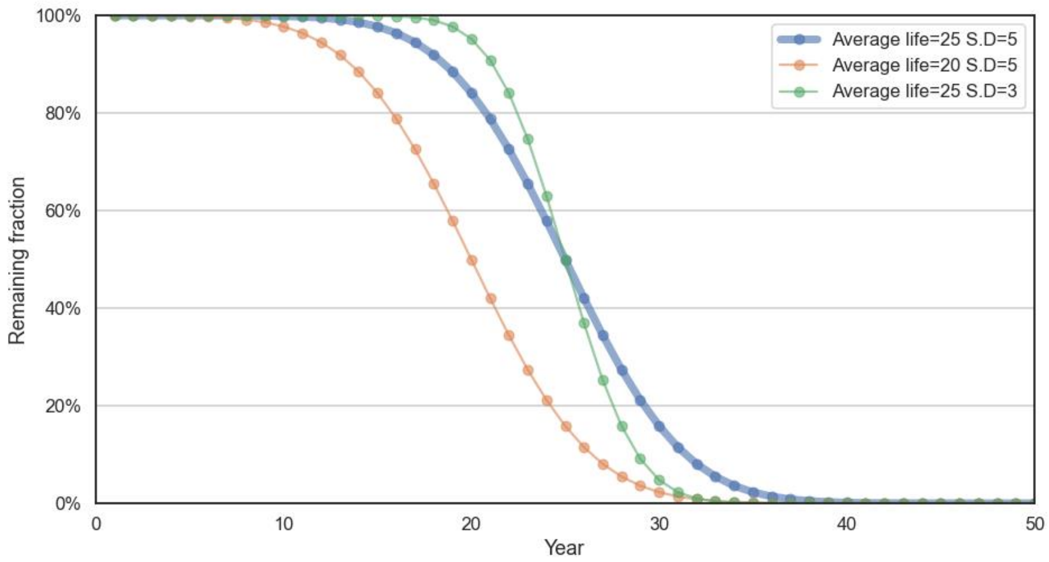

Figure S1. The survival curves in this study describe the probabilities that the previously installed capacities have not reached their lifetime at a certain point and are still operational. The average lifetime represents the time when half of the installed capacities are still operational. In our headline results, the average lifetime of wind projects is set to 25 years with a standard deviation of 5 years. In sensitivity analysis, we include two other sets of parameters: a) the same average lifetime (25 years), but with a smaller standard deviation of 3 years; b) the shorter average lifetime (20 years) with the same standard deviation of 5 years.

1.5 Sensitivity analysis

Our model outputs are based on a set of assumptions on variables: the lifetime distribution of power projects, the metal intensities for each component of e, array cable length for individual projects, and distance to main grids. The purpose is not to use our model to predict the future, but rather to explore quite different future scenarios of global development of electricity transmission infrastructure triggered by wind and solar PV generation and thus of metal requirement, and to understand key factors influencing the amount of required metal. A sensitivity analysis is thus performed for understanding where the major uncertainty may arise,

as well as assessing the impact of modeling assumptions on the simulation outcomes. We assess the impacts of lifetime parameters on outputs by applying a different set of normal distribution parameters. We also compare the headline outputs to alternative simulation outputs where the metal content of copper is assumed to be 10% less than the current level. In addition, we examine the effect of the cabling optimization of the inter-grids and project site selection on the amount of metal required by assuming that the array cable length of a project decreases by 10% and the distance to main grids increases by 10%. All alternative simulation processes are placed in the SDS scenario and only copper is tested.

2. Results

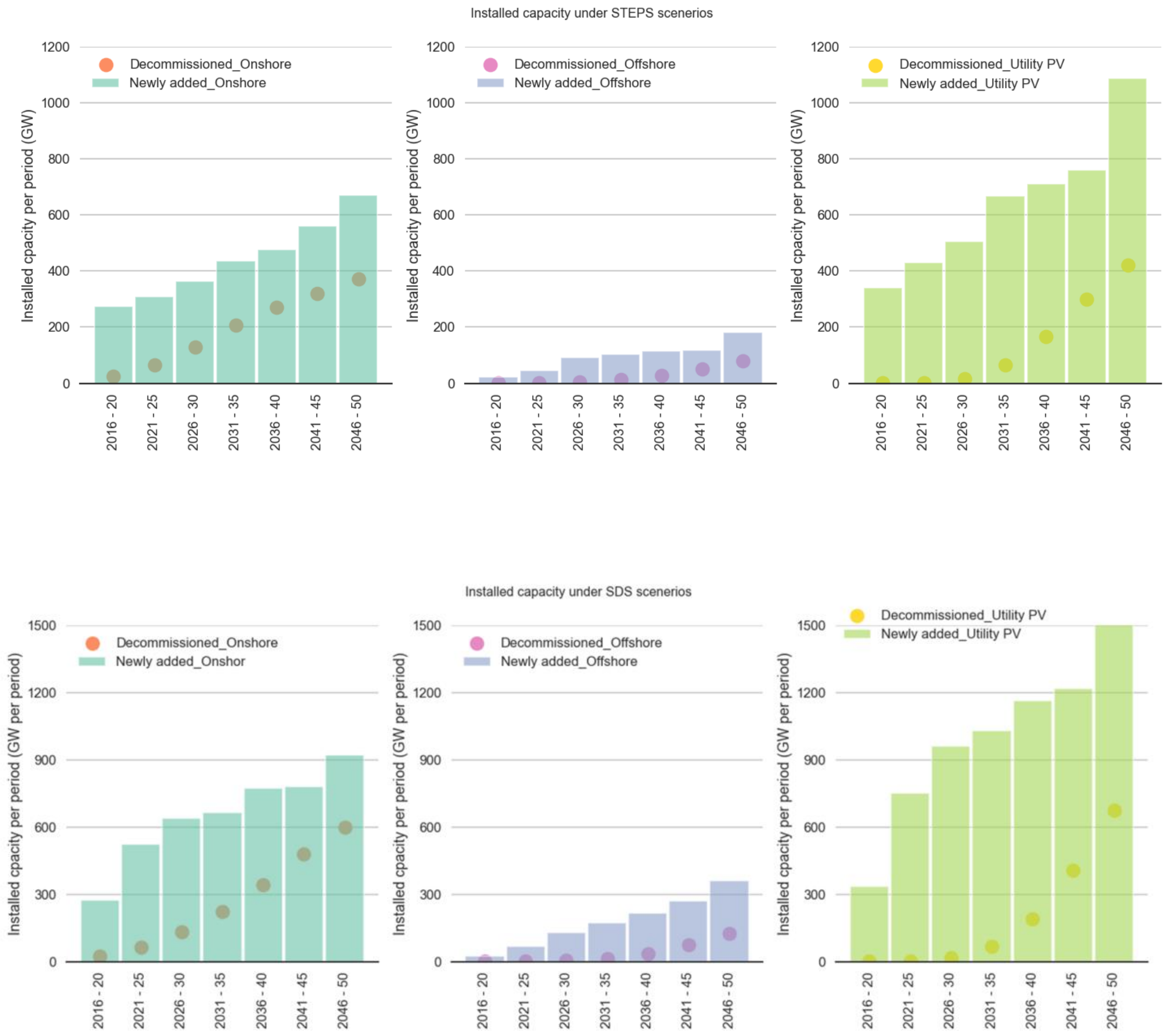

Figure S2. Newly installed capacities per period for utility-scale PV & wind (onshore and offshore) power under the STEPS and SDS scenario respectively.

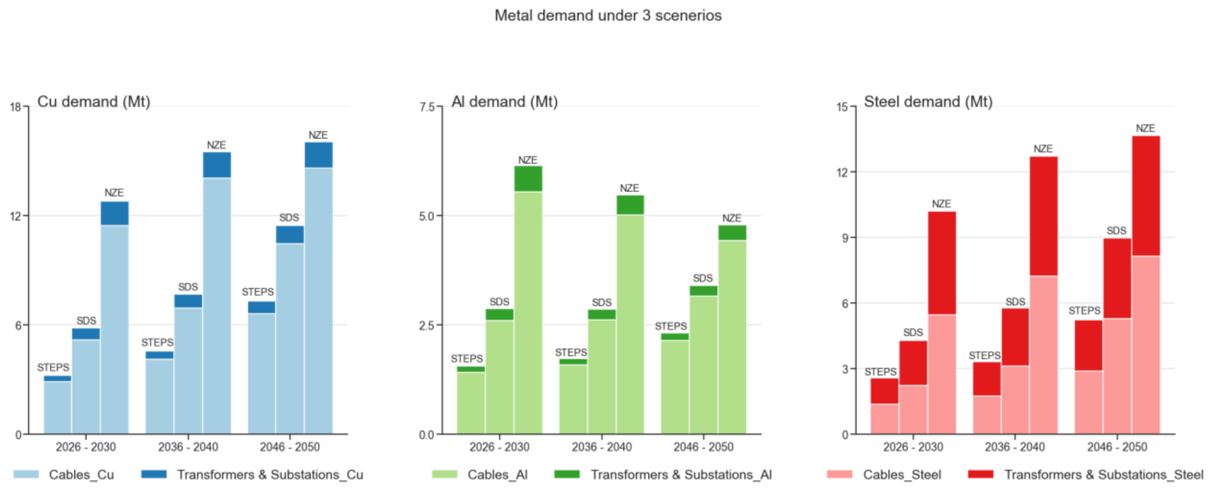

Figure S3. Comparison of metal demand per period for the electrical grid systems related to wind and utility-scale solar PV under three scenarios. Here light shades represent metals contained in cables, dark shades represent metals contained in main transformers and other electrical equipment.

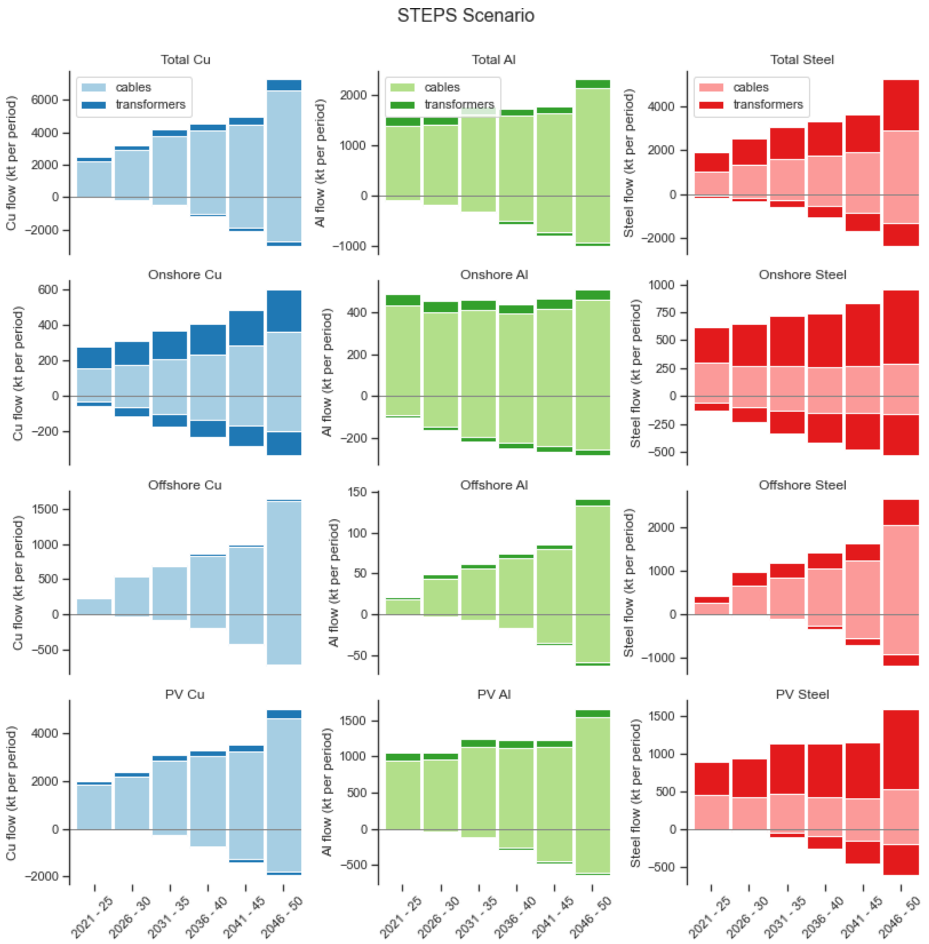

Figure S4. Metal demands (inflows) and corresponding decommissioned metal (outflows) for each period of newly built electrical grid systems associated with wind and utility-scale solar PV projects towards 2050 in the STEPS scenario by technology.

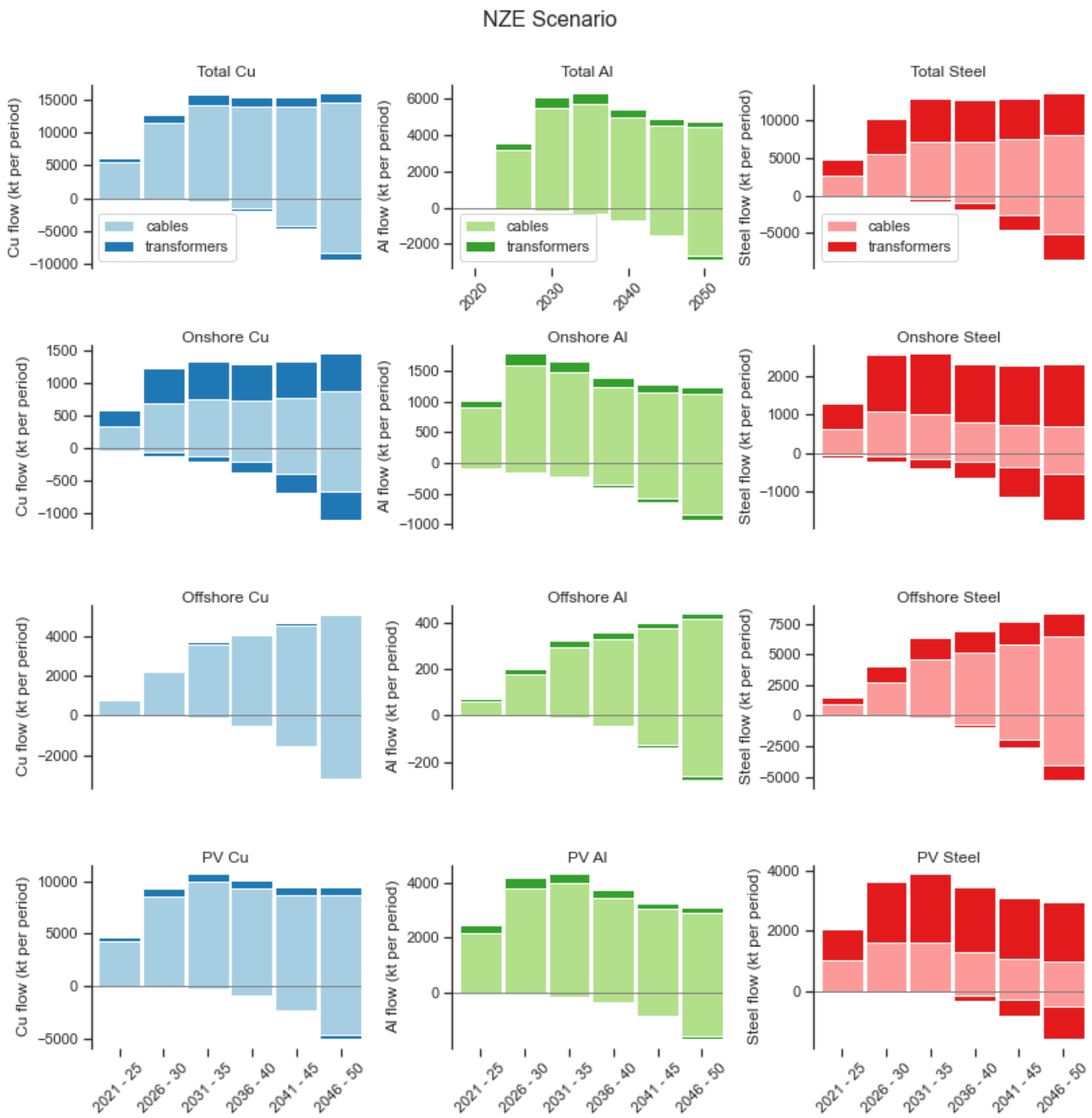

Figure S5. Metal demands (inflows) and corresponding decommissioned metal (outflows) for each period of newly built electrical grid systems associated with wind and utility-scale solar PV projects towards 2050 in the NZE scenario by technology.

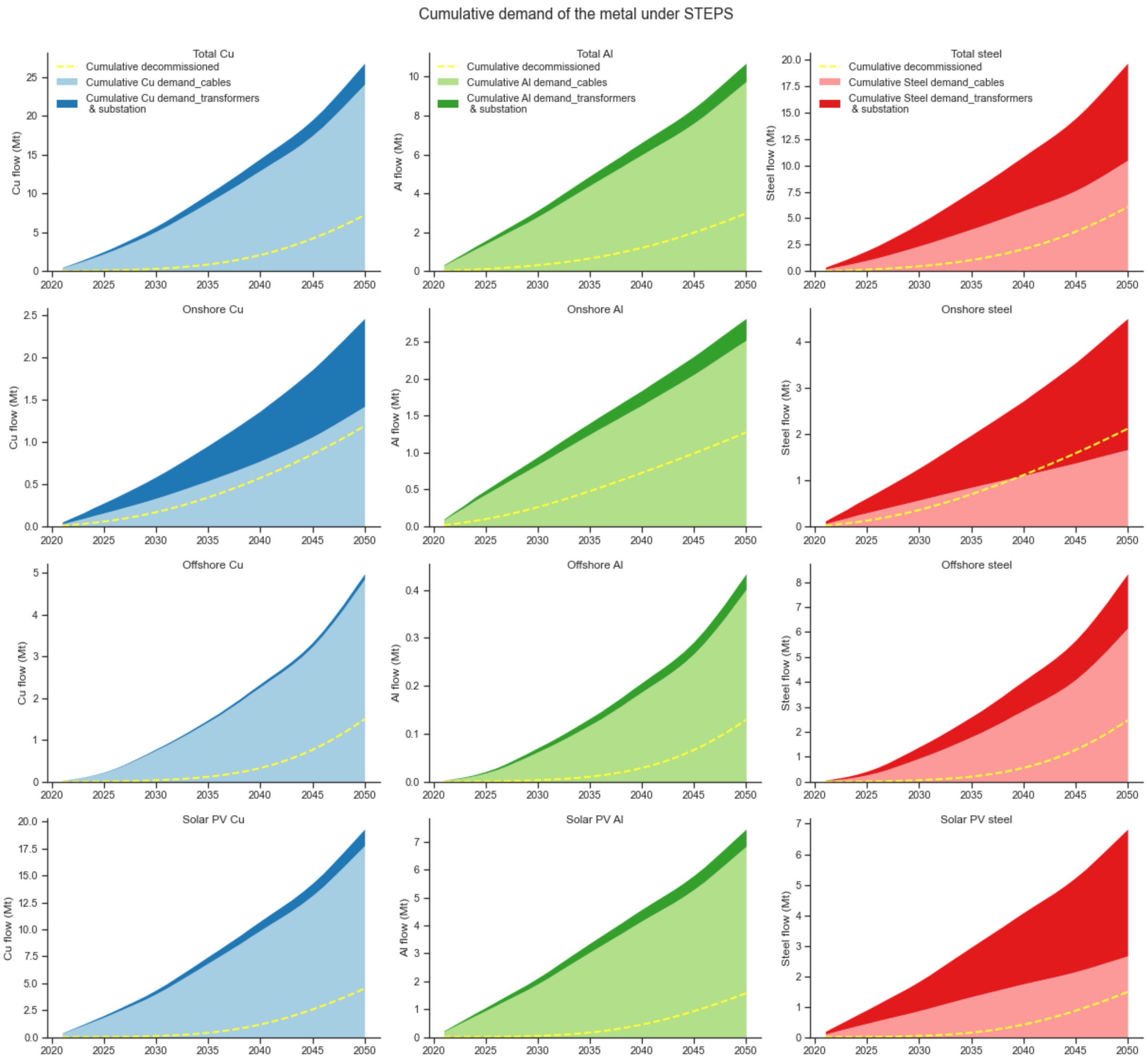

Figure S6. Cumulative metal demand and End-of-life (EOL) outflow for the dynamics of electrical grids accompanying wind and utility-scale solar projects over time by 2050 under the STEPS.

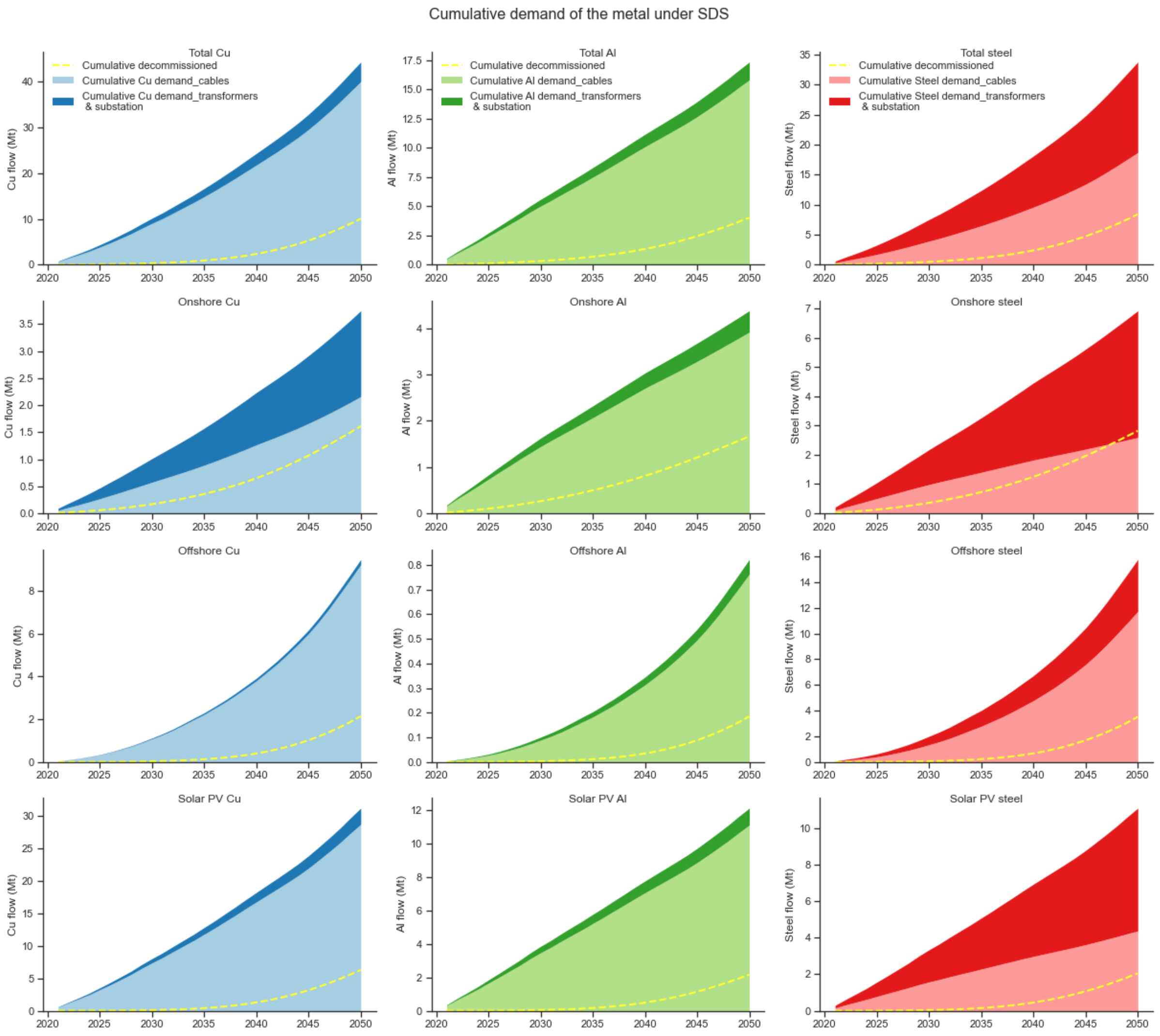

Figure S7. Cumulative metal demand and End-of-life (EOL) outflow for the dynamics of electrical grids accompanying wind and utility-scale solar projects over time by 2050 under the SDS.

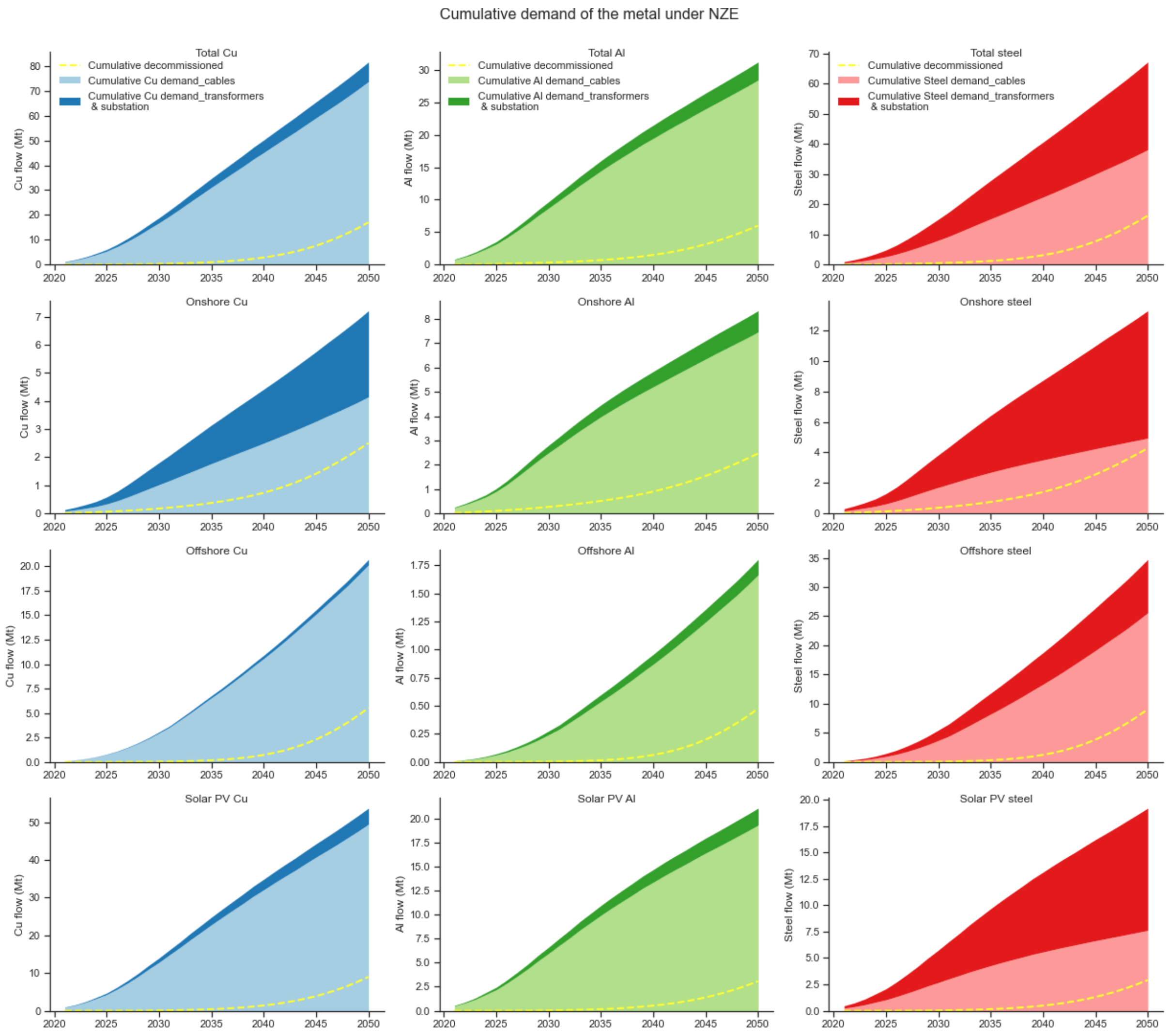

Figure S8. Cumulative metal demand and End-of-life (EOL) outflow for the dynamics of electrical grids accompanying wind and utility-scale solar projects over time by 2050 under the NZE scenario.

3. Uncertainties and sensitivity analysis

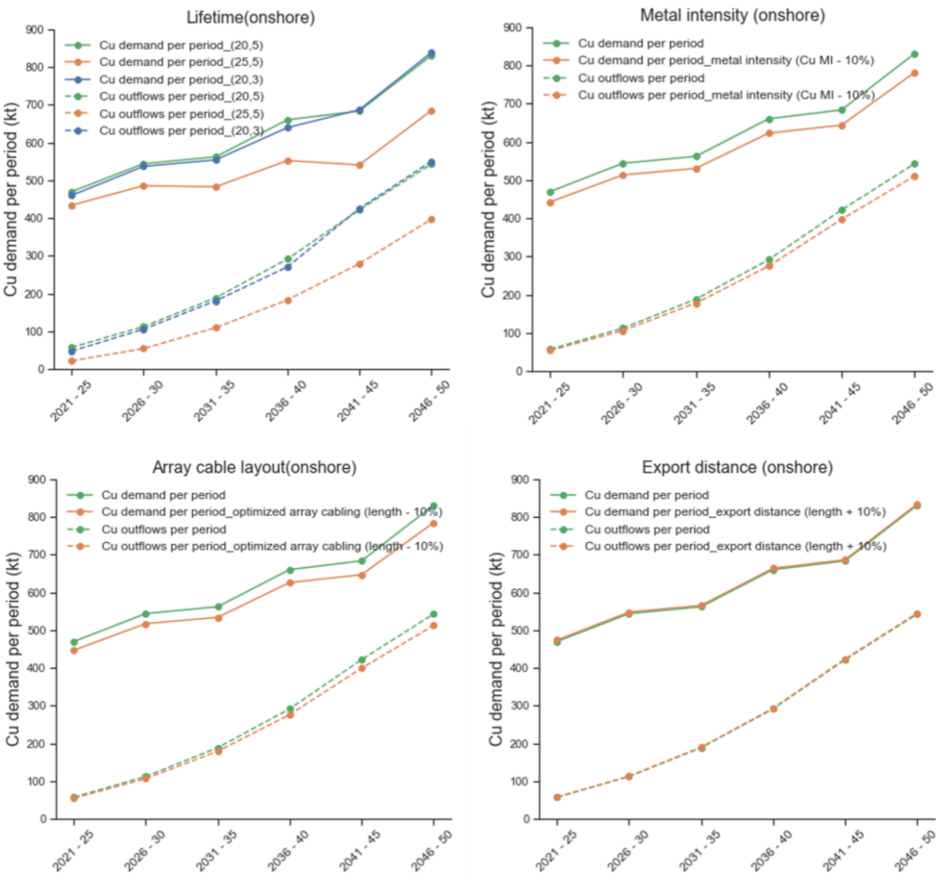

Figure S9. Impacts of the lifetime, metal content, array cable layout, and export distance on copper demand for onshore wind during 2021 - 2050 in the SDS.

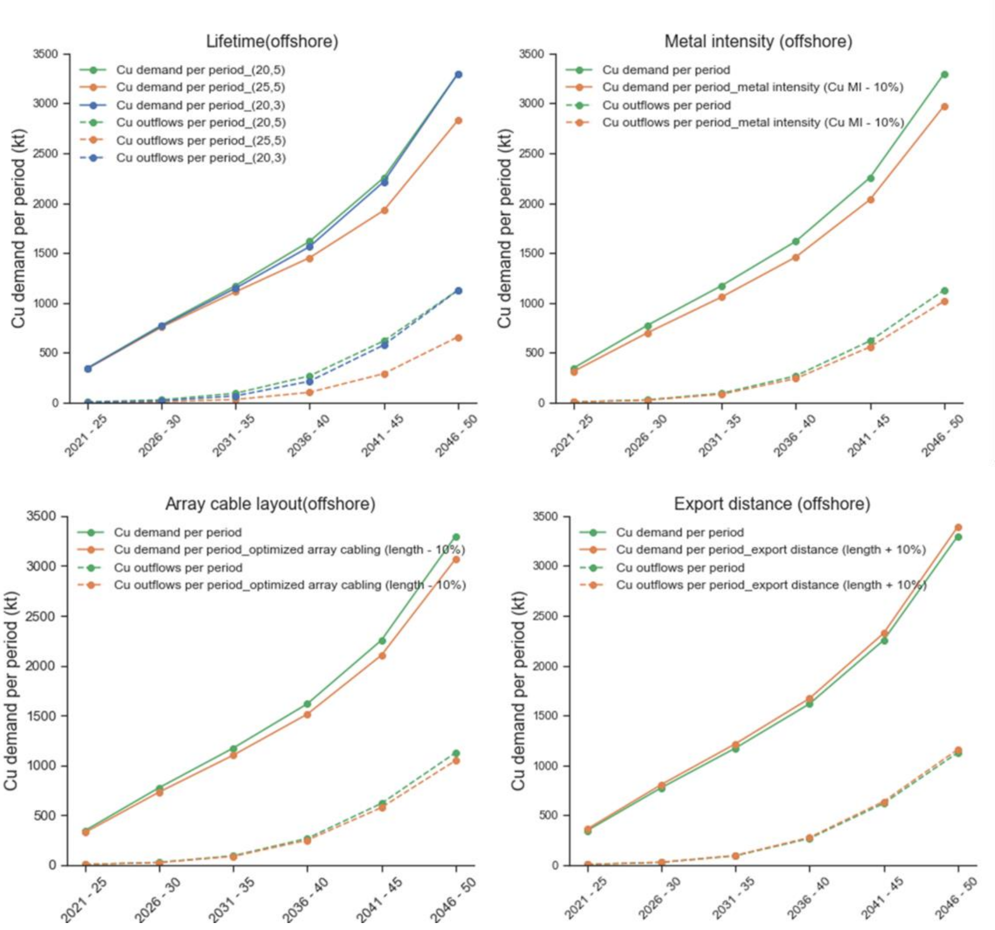

Figure S10. Impacts of the lifetime, metal content, array cable layout, and export distance on copper demand for offshore wind during 2021 - 2050 in the SDS.

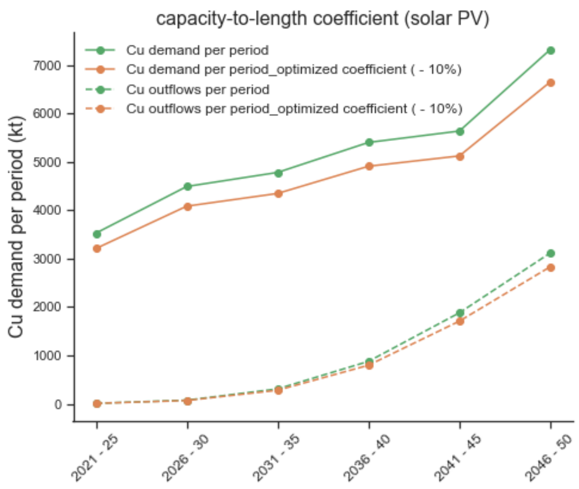

Figure S11. Impacts of length coefficient of infield cable on copper demand for solar PV during 2021 - 2050 in the SDS.

Copper flows contained in electrical grid systems for wind projects in the SDS scenario are selected to demonstrate the impacts of main uncertainties on modeling output. Figure 8 presents the results of model sensitivity analysis for onshore wind projects (see Figure S9 for offshore transmission lines). A longer average lifetime of renewable projects would reduce both the copper demand and outflows per period, as is expected. If the average lifetime of Normal lifetime distribution is extended to 25 years, copper demand per period will decrease to 685 kt per period by 2050, which is a about 18% drop compared to the original estimate. Meantime, as the average lifetime increases, the copper outflow also decreases, which keeps the difference between copper inflow and outflow almost unchanged. Besides, the smaller standard deviation parameter somewhat delays the required copper inflow, but after 2036, the effect is almost negligible. All of the above suggests that extending the life of the renewable program through technological advances could reduce overall future metal material requirements for building their corresponding transmission lines despite the almost unchanged difference between inflow and outflow.

Metal contents also have a significant impact on metal demand for power transmission lines. Grid operators have been trying to increase the share of cheaper aluminum conductors in submarine and underground power cables because of cost concerns. At the same time, the recent increasing use of DC power transmission systems will also reduce the metal content of power cables to a certain extent because compared to an AC power cable with a minimum of three wires, a DC power cable generally only uses two wires but has a greater power load capacity. Both trends indicate that technological developments are likely to reduce the metal content of power transmission lines. Following this trend, for onshore wind projects, a 10% reduction in copper content in cables would reduce copper demand per period by about 6% by 2050. For offshore wind, the copper demand per period would also fall by 10%. This also reminds us that we should improve the quality of information and data related to the metal content of

transmission cables and substations in the future, which will have a positive impact on the accuracy of the estimates.

The cable layout of inter-array grids and distance to interconnection points are also key factors in estimating metal demands. In the case of wind farms, different cabling layouts of the inter-array grid differ in total cable length, and thus power loss, generation efficiency, and costs, etc. Many studies have been conducted to find the optimal cable arrangement and topology of the inter-array system to find the optimal balance between increasing the power generation efficiency and reducing the required cable length[17,79,80]. The sensitivity analysis shows that if the total inter-array cable length is reduced by 10%, the copper demand associated with wind power projects would be reduced by 6% (onshore) and 7% (offshore) respectively. In addition, although the length of the export power cable related to the distance from the main network is likely to increase in the future, the increase in this parameter has little impact on the copper demand. These results further emphasize the importance of infield cable optimization from the perspective of metal resource demand.

The length coefficient of inter-array cable is a key consumption for estimating metal demand for utility-scale solar PV technology. The sensitivity analysis shows that if the length coefficient is reduced by 10%, the copper demand with solar PV projects would reduce by about 9%. This result also shows the robustness of the model.

References

(1) Cozzi; Gould; Bouckart; Crow; Kim. World Energy Outlook 2020. *Volksbl. (Bloemfontein, Print)*.
(2) Bouckaert, S.; Pales, A. F.; McGlade, C.; Remme, U.; Wanner, B.; Varro, L.; D'Ambrosio, D.; Spencer, T. Net Zero by 2050: A Roadmap for the Global Energy Sector. **2021**.
(3) European Regional Development Fund. *Future Energy Industry Trends*. https://northsearegion.eu/northsee/e-energy/future-energy-industry-trends/ (accessed 2021-10-18).
(4) Díaz, H.; Guedes Soares, C. Review of the Current Status, Technology and Future Trends of Offshore Wind Farms. *Ocean Eng.* **2020**, *209*, 107381.
(5) Enevoldsen, P.; Valentine, S. V. Do Onshore and Offshore Wind Farm Development Patterns Differ? *Energy for Sustainable Development* **2016**, *35*, 41–51.
(6) IEA. *IEA, Average awarded project size in utility-scale solar PV, Europe and emerging markets, 2013-2017*. https://www.iea.org/data-and-statistics/charts/average-awarded-project-size-in-utility-scale-solar-pv-europe-and-emerging-markets-2013-2017.
(7) Miller, A. Economics of Utility-Scale Solar in Aotearoa New Zealand.
(8) Musial, W. D.; Beiter, P. C.; Spitsen, P.; Nunemaker, J.; Gevorgian, V. *2018 Offshore Wind Technologies Market Report*; National Renewable Energy Lab.(NREL), Golden, CO (United States), 2019.
(9) Ramírez, L.; Fraile, D.; Brindley, G. Offshore Wind in Europe: Key Trends and Statistics 2019. **2020**.
(10) Beiter, P. C.; Tian, T.; Nunemaker, J.; Musial, W. D.; Lantz, E. J.; Gevorgian, V.; Spitsen, P. *2017 Offshore Wind Technologies Market Update*; National Renewable Energy Lab.(NREL), Golden, CO (United States), 2018.
(11) Garrett, P.; Ronde, K. Life Cycle Assessment of Electricity Production from an Onshore V126-3.3 MW Wind Plant. *Vestas Wind Systems A/S* **2014**.
(12) Garrett, P.; Ronde, K. Life Cycle Assessment of Electricity Production from an V126-3.45 Onshore Wind Plant Vestas. *Vestas Wind Systems A/S* **2017**.
(13) Mondol, J.; Jacob, G. Commercial Scale Solar Power Generation (5MW to 50 MW) and Its Connection to Distribution Power Network in the United Kingdom. *Journal of Solar Energy Research Updates* **2018**, *5*, 25–38.
(14) Sward, J. A.; Siff, J.; Gu, J.; Max Zhang, K. Strategic Planning for Utility-Scale Solar Photovoltaic Development – Historical Peak Events Revisited. *Applied Energy*. 2019, pp 1292–1301. https://doi.org/10.1016/j.apenergy.2019.04.178.
(15) Fischetti, M.; Pisinger, D. Optimal Wind Farm Cable Routing: Modeling Branches and Offshore Transformer Modules. *Networks* **2018**.
(16) El Mokhi, C.; Addaim, A. Optimization of Wind Turbine Interconnections in an Offshore Wind Farm Using Metaheuristic Algorithms. *Sustain. Sci. Pract. Policy* **2020**, *12* (14), 5761.
(17) Pillai, A. C.; Chick, J.; Johanning, L.; Khorasanchi, M.; de Laleu, V. Offshore Wind Farm Electrical Cable Layout Optimization. *Eng. Optim.* **2015**, *47* (12), 1689–1708.
(18) Kaiser, M. J.; Snyder, B. Offshore Wind Energy Installation and Decommissioning Cost Estimation in the Us Outer Continental Shelf. *US Dept. of the Interior, Bureau of Ocean Energy Management, Regulation and Enforcement, Herndon, VA TA&R* **2010**, *648*.
(19) Schachner, J. *Power Connections for Offshore Wind Farms*; na, 2004.
(20) Evans, S. *The biggest solar power plants in the world*. https://www.power-technology.com/features/the-worlds-biggest-solar-power-plants/ (accessed 2021-10-20).

(21) *White paper on solar DC cables*. https://renewablewatch.in/2018/07/09/white-paper-solar-dc-cables/ (accessed 2021-10-20).
(22) Satpathy, R. K.; Pamuru, V. *Solar PV Power: Design, Manufacturing and Applications from Sand to Systems*; Academic Press, 2020.
(23) Borup, U.; Grau, H.; Lave, B. String Inverters for PV Power Plants. **2009**. https://doi.org/10.4229/24thEUPVSEC2009-5BV.2.44.
(24) Wind Turbine Grid Connection and Interaction.pdf.
(25) Jingwei, Y. Analysis of Wind Farm' S Connection Modes , Grid Connection and Operation Modes. **2012**.
(26) Alassi, A.; Bañales, S.; Ellabban, O.; Adam, G.; MacIver, C. HVDC Transmission: Technology Review, Market Trends and Future Outlook. *Renewable Sustainable Energy Rev.* **2019**, *112*, 530–554.
(27) Foltyn, S. R.; Civale, L.; Macmanus-Driscoll, J. L.; Jia, Q. X.; Maiorov, B.; Wang, H.; Maley, M. Materials Science Challenges for High-Temperature Superconducting Wire. *Nat. Mater.* **2007**, *6* (9), 631–642.
(28) Jin, J. X.; Xin, Y.; Wang, Q. L.; He, Y. S.; Cai, C. B.; Wang, Y. S.; Wang, Z. M. Enabling High-Temperature Superconducting Technologies Toward Practical Applications. *IEEE Trans. Appl. Supercond.* **2014**, *24* (5), 1–12.
(29) Baring-Gould, I. Offshore Wind Plant Electrical Systems. 2014.
(30) DNV KEMA Renewables, Inc. *Appendix D Substation and Cable Route Design Report*; DNV KEMA Renewables, Inc., 2014.
(31) Hans de Boer, A. van der H. *Inventory Offshore Wind Test Sites Demand & Supply in the Netherlands*; BLIX Consultancy.
(32) TenneT. *66 kV Systems for Offshore Wind Farms*; 113799-UKBR-R02, Rev. 2; TenneT, 2015.
(33) Birkeland, C. *Assessing the life cycle environmental impacts of offshore wind power generation and power transmission in the North Sea*. https://ntnuopen.ntnu.no/ntnu-xmlui/bitstream/handle/11250/257062/440527_FULLTEXT01.pdf?sequence=1&isAllowed=y (accessed 2021-05-25).
(34) Prysmian Group. *66 kV Submarine Cable Systems for Offshore Wind*.
(35) Neumann, A. P.; Mulroy, M. J.; Ebden, C. The Use of 66kV Technology for Offshore Wind Demonstration Sites. *3rd Renewable Power Generation Conference (RPG 2014)*. 2014. https://doi.org/10.1049/cp.2014.0832.
(36) Ferguson, A.; de Villiers, P.; Fitzgerald, B.; Matthiesen, J. Benefits in Moving the Inter-Array Voltage from 33 kV to 66 kV AC for Large Offshore Wind Farms. *EWEA 2012* **2012**.
(37) Kaltenborn, U.; Coors, P.; Pietsch, R.; Steiner, T. On-Site Testing of 66 kV Subsea Array Cables for off-Shore Windfarms. AIM June 3, 2019. https://doi.org/10.34890/851.
(38) de la Vieter, D. I. M. *33/66 kV Inter-Array Cables for Dutch Offshore Wind Farms*; BLIX Consultancy BV, 2015.
(39) Boone, W.; Sonderen, C. Copper in Comparison with Aluminium as Common Material in Conductors of Lv and Mv Cables. In *Proceedings of 23rd International Conference on Electricity Distribution, Lyon, France*; 2015; pp 26–25.
(40) Sven MUELLER-SCHUETZE, Heiner OTTERSBERG, Carsten SUHR, Ingo KRUSCHE, Norddeutsche Seekabelwerke. Development of Submarine MV-AC Power Cable with Aluminum Conductor; 9th International Conference on Insulated Power Cables, 2015.
(41) Worzyk, T.; Långström, S. Use of Aluminum Conductors in Submarine Power Cables. In *9th International Conference on Insulated Power Cables, Versailles, Technical Paper, Versailles, France*; 2015.

(42) Ankit Gupta, A. S. B. *Offshore Wind Cable Market size to exceed $3 billion by 2026*. https://www.gminsights.com/pressrelease/offshore-wind-cable-market (accessed 2021-11-11).
(43) *Aluminium vs. Copper*. https://www.icf.at/news/icf-news-78-2017-apr/aluminium-vs-copper/ (accessed 2021-11-11).
(44) EirGrid. *Functional Specification 110/220/400kV Submarine Cables*; 2020.
(45) Weerheim, R. Development of Dynamic Power Cables for Commercial Floating Wind Farms. *Literature assignment* **2018**.
(46) Wang, L.; Wu, J.; Tang, Z.; Wang, T. An Integration Optimization Method for Power Collection Systems of Offshore Wind Farms. *Energies* **2019**, *12* (20), 3965.
(47) NexansGermany. NEXANS TO SUPPLY E.ON WITH SUBMARINE CABLES FOR THE ARKONA OFFSHORE WIND FARM. 2016.
(48) *Beatrice Offshore Wind Farm Consent Plan*; LF000005-PLN-179 Rev 2.0; 2017.
(49) Zouraraki, M.; Kvarts, T.; Østerø, R.; Page, T.; Hjerrild, J.; Vilhelmsen, M. A. Hornsea Projects 1 and 2-Design and Optimisation of the Cables for the World Largest Offshore Wind Farms. In *International Conference Insulated Power Cables (Jicable19), Versailles, A2-6. http://www. jicable. org/TOUT_JICABLE_FIRST_PAG E/2019/2019-F2-6_page1. pdf*; 2019.
(50) Arvesen, A.; Christine; Birkeland; Hertwich, E. G. The Importance of Ships and Spare Parts in LCAs of Offshore Wind Power. *Environmental Science & Technology*. 2013, pp 2948–2956. https://doi.org/10.1021/es304509r.
(51) Arvesen, A.; Hertwich, E. G. Assessing the Life Cycle Environmental Impacts of Wind Power: A Review of Present Knowledge and Research Needs. *Renewable Sustainable Energy Rev.* **2012**, *16* (8), 5994–6006.
(52) OrientCable. *Submarine Cable*; Orient cable, 2011.
(53) *XLPE Submarine Cable Systems Attachment to XLPE Land Cable Systems User´s Guide*; {ABB's high voltage cable unit in Sweden}, 2010.
(54) Nexans. *Technical Data for the 36kV Submarine*; Nexans, 2013.
(55) INNOSEA. *Review of the State of the Art of Dynamic Cable System Design*; D3.1; 2020.
(56) ZTTCable. *SUBMARINE CABLE*; ZTTCable, 2013.
(57) Fisheries Technical Working Group. *Offshore Wind Submarine Cabling Overview*; Tetra Tech, Inc., 2021.
(58) Worzyk, T.; Långström, S. Use of Aluminum Conductors in Submarine Power Cables, 2015.
(59) Nexans. *INTEGRATED CABLE SOLUTIONS FOR OFFSHORE WIND DEVELOPMENT*; 2018.
(60) EIA. *Assessing HVDC Transmission for Impacts of Non-dispatchable Generation*; EIA, 2018.
(61) Neart na Gaoithe Offshore Wind Ltd. *Chapter 4 Project Description*; UK02-0504-0741-MRP-OFFSHORE_EIAR-RPT-A2; 2018.
(62) A Friday, M. L. *Economic Analysis of Large Submarine Cables*; 2016-0350; 2016.
(63) LS cable Ltd; Energy Cable & System Bussiness Group. LS Cable's Submarine & Umbilical Cable System.
(64) Corporativa, I. *What is wind energy, how is it converted into electricity and what are its advantages?*. https://www.iberdrola.com/environment/renewables-energy-wind-power (accessed 2021-11-11).
(65) Nexans, S. A. SA 6--36 kV Medium Voltage Underground Power Cables: XLPE Insulated Cables. 2009.
(66) NexansFrance. *60-500 kV High Voltage Underground Power Cables*; 2011.
(67) Sphera Solutions, Inc. *LCA of a Nordex Windfarm with Delta4000 Turbines*; 2020.

(68) Jorge, R. S.; Hawkins, T. R.; Hertwich, E. G. Life Cycle Assessment of Electricity Transmission and Distribution—part 1: Power Lines and Cables. *The International Journal of Life Cycle Assessment*. 2012, pp 9–15. https://doi.org/10.1007/s11367-011-0335-1.
(69) Arvesen, A.; Hertwich, E. G. Environmental Implications of Large-Scale Adoption of Wind Power: A Scenario-Based Life Cycle Assessment. *Environ. Res. Lett.* **2011**.
(70) Jorge, R. S.; Hertwich, E. G. Environmental Evaluation of Power Transmission in Norway. *Appl. Energy* **2013**, *101*, 513–520.
(71) Wendt, V. Underground Cables in Europe: Overview, 2017.
(72) Nexans. POWER & DATA CABLE INFRASTRUCTURES TO BOOST SOLAR ENERGY COMPETITIVENESS, RELIABILITY AND SUSTAINABILITY. 2017.
(73) Simpson, D. Basics of Medium Voltage for PV Power Plant AC Collection Systems. 2013.
(74) HELUKABE. Cables and Cable Systems for Photovoltaic Installations. 2011.
(75) GeneralCable. Building Wire.pdf. 2017.
(76) Harrison, G. P.; Maclean, E. (ned) J.; Karamanlis, S.; Ochoa, L. F. Life Cycle Assessment of the Transmission Network in Great Britain. *Energy Policy* **2010**, *38* (7), 3622–3631.
(77) Jorge, R. S.; Hawkins, T. R.; Hertwich, E. G. Life Cycle Assessment of Electricity Transmission and Distribution—part 2: Transformers and Substation Equipment. *Int. J. Life Cycle Assess.* **2012**, *17* (2), 184–191.
(78) ABB. Power Transformers.pdf.
(79) Tifroute, M.; Bouzahir, H. Optimization for Onshore Wind Farm Cable: Connection Layout Using ACO-AIA Algorithm. *Matematika* **2019**, *35* (1).
(80) Cui, Y.; Zhang, K.; Zhang, Z.; Yang, S.; Xu, H.; Liu, Y. Optimization of Electric System for Offshore Wind Farm Based on Lightweight Substation. *IOP Conf. Ser. Earth Environ. Sci.* **2018**, *186*, 012023.
